# Biosilica from Agricultural Residues for Hazardous Contaminant Removal and Soil Remediation: Synthesis Routes, Structure–Property Relationships, and Environmental Relevance

**DOI:** 10.3390/ijms27219737

**Published:** 2026-10-30

**Authors:** Veronica Latini, Agata Wawrzyńczak, Agnieszka Feliczak-Guzik

**Affiliations:** Department of Applied Chemistry, Faculty of Chemistry, Adam Mickiewicz University in Poznań, Uniwersytetu Poznańskiego 8, 61-614 Poznań, Poland; verlat@st.amu.edu.pl

**Keywords:** biosilica, agricultural residues, adsorbents, hazardous contaminants, soil remediation, water treatment

## Abstract

The conversion of agricultural residues into functional silica materials offers opportunities to combine waste valorization with environmental remediation. This review critically examines biosilica production from rice husk and straw, corn, sugarcane, wheat, and selected fruit and potato wastes, focusing on sol–gel processing, hydrothermal synthesis, and acid leaching. To address feedstock heterogeneity, reported silicon and silica contents are distinguished according to their analytical basis, including biomass and ash fractions. Synthesis routes are compared using available quantitative indicators, including silica yield, purity, specific surface area, and processing conditions, while accounting for differences in reporting practices and incomplete datasets. Particular attention is given to the relationships between precursor composition, synthesis parameters, surface chemistry, and contaminant-removal performance. The analysis highlights the importance of accessible surface sites, functional groups, and contaminant speciation, demonstrating that specific surface area alone cannot predict adsorption efficiency. Although biosilica shows potential for water treatment and soil remediation, practical implementation remains constrained by feedstock variability, limited testing under realistic conditions, regeneration challenges, and uncertainties regarding long-term safety. The review identifies key research priorities for developing reproducible, economically viable, and environmentally sustainable biosilica technologies.

## 1. Introduction

In recent years, silica (SiO_2_) has found increasingly widespread applications in environmental protection, biomedicine, agriculture, and the chemical industry [1,2,3,4,5,6,7]. Among these applications, the use of silica in hazardous contaminant management has attracted particular attention because silica-based materials combine a high specific surface area, tunable porosity, thermal stability, and readily modifiable surfaces [1,2,5,8,9,10,11].

These features make silica attractive for the adsorption, immobilization, and controlled interaction of hazardous species in water, soil, and waste-derived matrices [1,8,9,10,11]. In environmental systems, silica has been investigated for the removal of dyes, heavy metals, nutrients, pesticides, and other problematic pollutants from aqueous media and for improving the physicochemical performance of contaminated or degraded soils [1,3,6,7,8,9,10,11]. At the same time, the growing demand for silica, together with the high energy intensity and cost of conventional production routes, has encouraged the search for more sustainable technological solutions [1,8,9,10,11]. In particular, methods based on biomass valorization enable biosilica production from renewable agricultural residues, such as rice husks, straw, and sugarcane waste, while offering opportunities to reduce waste accumulation and the environmental impact of silica production [1,8,9,10,11].

Such an approach links waste valorization with the development of functional materials for pollution control and is therefore aligned with circular-economy and sustainable-development principles [1,2,3,4,5,6,7,8,9,10,11].

Silicon is the second most abundant element in the Earth’s crust after oxygen [7]. Despite its natural abundance, specialty silica materials are predominantly produced using synthetic methods, including precipitation, sol–gel processing, and high-temperature synthesis routes [1,12,13,14,15,16].

Although these methods are well established and allow control over the properties of the final product, they may involve high energy consumption, the use of aggressive chemical reagents, and the generation of substantial waste streams. Among silicate-based materials, zeolites represent an important class of crystalline microporous aluminosilicates characterized by well-defined pore structures, ion-exchange capacity, and shape-selective properties. These characteristics contribute to their industrial significance, particularly in heterogeneous catalysis, petrochemical processing, and environmental remediation [10,17,18]. Zeolite-based materials have also demonstrated considerable potential for contaminant removal from aqueous solutions, including the adsorption of heavy metals such as cadmium [18,19].

**Figure 1:**
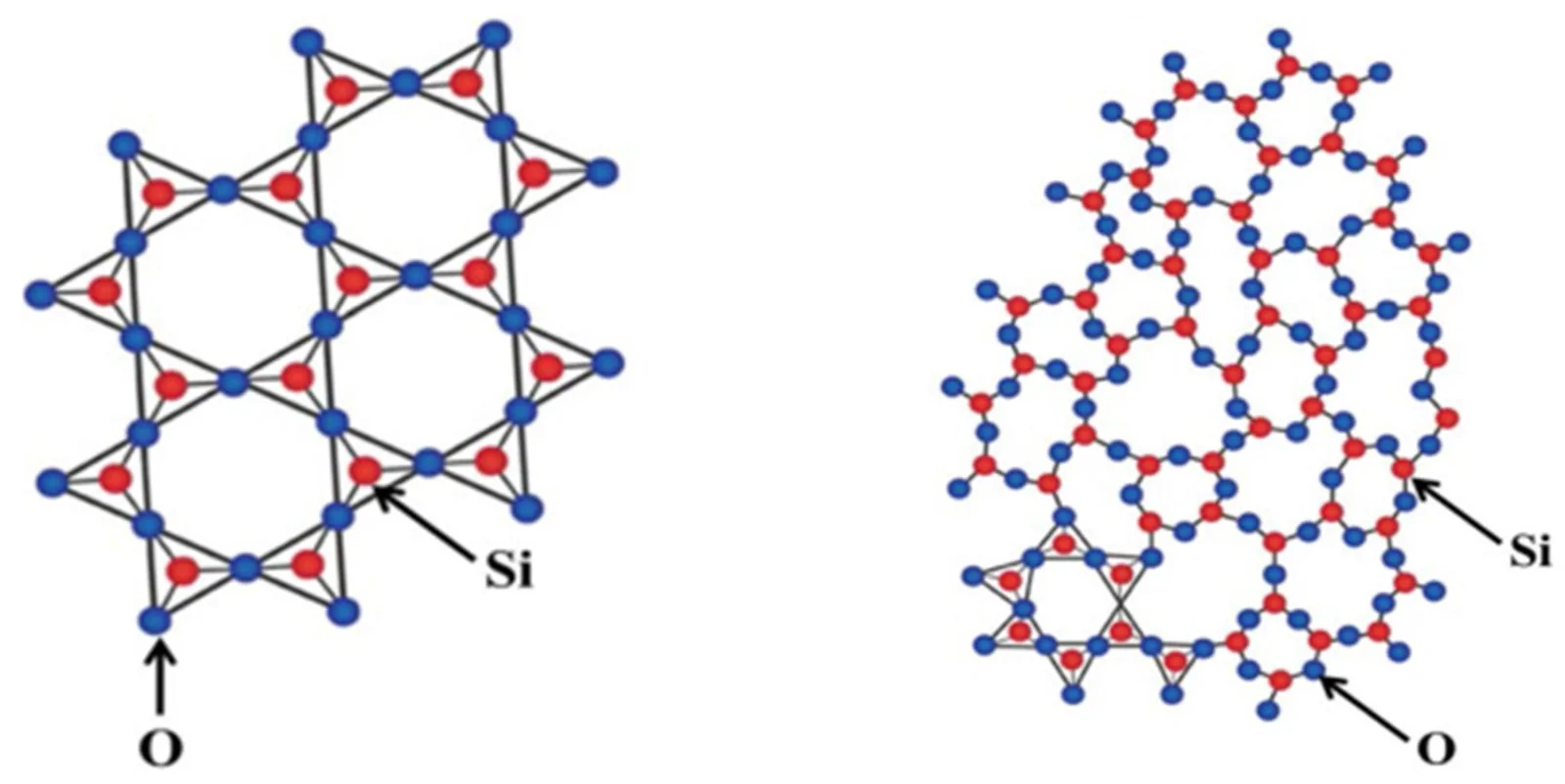
Structure of crystalline and amorphous silica. Adapted from Ref. [30] with permission from Springer.

Silica can be obtained from various natural sources such as quartz sand, silicate rocks, clays, or volcanic soils, as well as from plant material, in which its content usually ranges from 0.1% to 10% [1,20,21,22,23]. In plant organisms, silica occurs mainly in the form of phytoliths, performing structural and protective functions [20,21,22,23]. Depending on the technological process and synthesis conditions, silica may be obtained in amorphous or crystalline form (Figure 1) [1,12,13,14,15,16,17,24,25,26,27]. 

The amorphous form, characterized by higher reactivity and a high specific surface area, is particularly valuable in technological and environmental applications, whereas crystalline forms such as quartz, tridymite, or cristobalite exhibit greater structural stability and are used mainly in construction materials and in the ceramic industry [12,13,14,15,16,17,20,21,22,23,24,25,26,27,28,29]. From the perspective of hazardous-materials research, the distinction between amorphous and crystalline forms is also important because it affects reactivity, sorption performance, environmental persistence, and the safety profile of the final material during production and application [1,12,13,14,15,16,17,20,21,22,23,24,25,26,27,28,29].

Studies suggest that plant-derived silica exhibits significant advantages compared with synthetic and mineral materials, especially in terms of energy consumption and environmental impact. Its production can take place under milder conditions, with lower energy consumption and without the need to use complex apparatus and expensive reagents. Such processes often involve the extraction of silicon-containing species from biomass using alkaline solutions, followed by silica precipitation through acidification, which can yield materials with high purity and a well-developed porous structure.

At the same time, the use of agricultural waste as a feedstock fits the circular-economy concept, making it possible to reduce the amount of waste and decrease dependence on conventional silica production. Such an approach also increases the added value of agricultural by-products and may contribute to the development of more sustainable local feedstock supply chains.

Despite the growing number of studies on biosilica derived from agricultural residues, a comprehensive assessment linking feedstock type and synthesis conditions with physicochemical properties and subsequent environmental performance is still needed [21,30,31,32,33,34,35,36,37,38,39,40]. Therefore, this review aims to summarize the main agricultural sources of biosilica, compare the most relevant synthesis routes, and evaluate how processing conditions influence material properties relevant to hazardous contaminant removal and soil remediation. Particular attention is given to structure–property relationships and to the environmental relevance of the resulting materials.

Previous publications have examined biomass-derived silica from different perspectives. Prabha et al. [30] reviewed plant-derived silica nanoparticles and composites, primarily addressing biomedical, sensing, and energy-storage applications, whereas Karande et al. [31] focused on green synthesis strategies. Errington et al. [32] evaluated the environmental implications of conventional and biomass-derived silica production using life-cycle assessment. Other studies have addressed nanosilica extraction from agricultural residues [33] and its applications in asphalt materials [35]. Although these publications provide valuable insights into production methods, sustainability, and selected applications, they do not comprehensively integrate material characteristics with contaminant-specific remediation performance. The novelty of the present review lies in establishing a structure–property–performance perspective that connects agricultural feedstock selection and processing conditions with adsorption mechanisms, contaminant immobilization, and soil remediation. Particular attention is given to regeneration potential, long-term environmental safety, and technological limitations affecting practical implementation. This approach enables the identification of critical research gaps and provides a basis for evaluating the applicability of biosilica in environmental technologies. In this review, the term “biosilica” is used broadly to describe silica obtained from biological or biomass-derived sources, particularly agricultural residues [1,32]. In contrast, “nanosilica” and “silica nanoparticles” refer to silica materials produced at the nanoscale [30,31]. Thus, “biosilica” primarily describes the origin of the silica, whereas “nanosilica” or “silica nanoparticles” describe its dimensional scale. Accordingly, these terms are not fully interchangeable, since biomass-derived silica may occur in both nanoscale and larger particulate forms.

### Literature Search Strategy

**Scheme 1:**
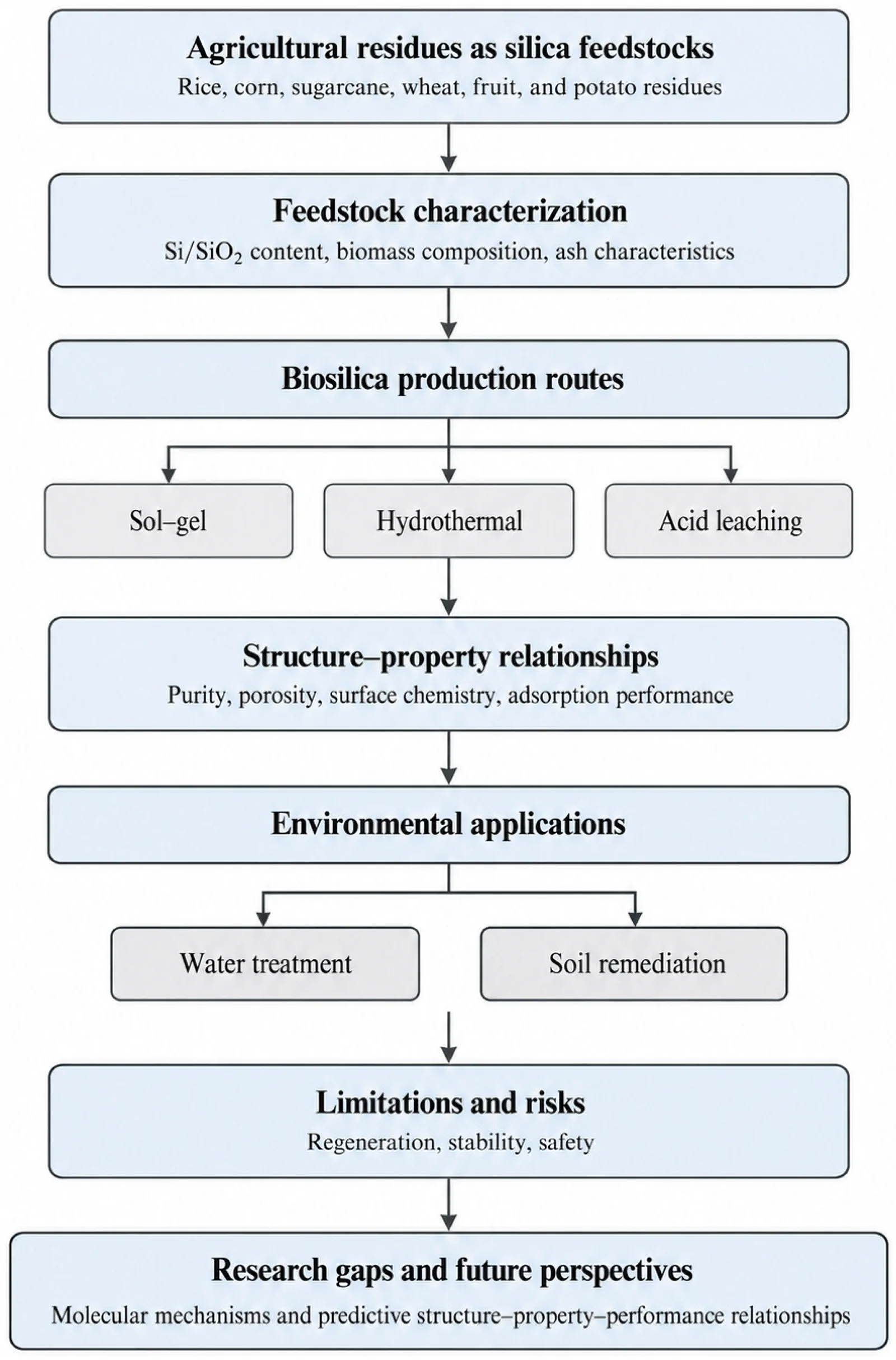
Schematic overview of the structure and main topics addressed in this review (prepared by the authors).

The literature included in this review was identified using the Scopus, Web of Science, and Google Scholar databases. Searches were performed using combinations of terms related to biosilica, agricultural residues, synthesis methods, material characterization, contaminant removal, and soil remediation. Representative keywords included “biosilica”, “biogenic silica”, “biomass-derived silica”, “agricultural waste silica”, “rice husk silica”, “sol-gel”, “acid leaching”, “hydrothermal synthesis”, “adsorption”, “heavy metals”, “dyes”, “pharmaceuticals”, and “soil remediation”. Peer-reviewed articles and reviews directly relevant to the scope of the manuscript were prioritized. Studies unrelated to silica-containing materials or not providing information relevant to biosilica production, characterization, or environmental performance were excluded. Recent publications were preferentially considered, while older studies were retained when they provided fundamental or methodologically relevant information. The overall structure and main thematic areas of this review are illustrated in Scheme 1.

## 2. Phytogenic Silica in Plants as a Source of Functional Materials

Silica occurring in plants is referred to as phytogenic silica and is located both inside cells (in the cell wall and in the cell lumen) and in intercellular spaces and extracellular layers, including epidermal tissues [23,41,42]. Its distribution within plants depends on the species, developmental stage, and environmental conditions [41,42,43].

Structures of phytogenic silica present in intercellular spaces and extracellular layers are characterized by relatively low mechanical strength and are susceptible to degradation, which makes them undergo relatively rapid transformations in the soil environment [41,44,45,46]. In contrast, silica deposits located within cell walls and cell lumina exhibit greater stability and resistance to environmental factors such as changes in pH, moisture, and microbial activity [41,44,46].

As a result, they may survive in soil for long periods, reaching hundreds or even thousands of years, in the form of microscopic structures known as phytoliths. These structures are not only an important element of the silicon cycle in nature [45,46], but also a valuable tool in paleobotanical studies and the reconstruction of past environmental conditions [42,47,48].

Silicon in soils occurs predominantly in silicate minerals and silica phases, whereas dissolved silicon in soil solution is present mainly as monosilicic acid (H_4_SiO_4_), with oligomeric and polymeric species occurring depending on solution conditions. Among them, monosilicic acid is the form directly assimilable by plants because it is soluble in water and easily transported in the soil solution. However, its concentration in soil is usually low and depends on many factors such as pH, temperature, biological activity, and the mineral composition of the soil. Polysilicic acid is formed as a result of polymerization processes of monosilicic acid, which may be initiated, among other things, by acidification of silicate solutions such as sodium silicate using mineral acids. This process leads to the formation of silica structures with varying degrees of condensation, which may eventually form gels or solid materials.

Organosilicon compounds, in turn, are formed as a result of interactions of silicon with soil organic matter and may constitute an important reservoir of this element, although their availability to plants is limited [49,50,51,52,53].

Silicon is taken up by plant roots primarily in the form of monosilicic acid (H_4_SiO_4_), the predominant soluble and bioavailable silicon species in soil solution [51,54,55,56].

Silicon uptake and transport involve both passive and transporter-mediated processes, including the activity of specialized membrane proteins such as Lsi1 (Low silicon 1), which facilitates silicic acid influx, and Lsi2 (Low silicon 2), which mediates its efflux from cells [55,56]. After uptake by the root system, silicon is transported with water through the xylem, and the transpiration process promotes its concentration in the aboveground parts of the plant [54,55,56,57]. As a result of these processes, silica condenses and precipitates in the form of amorphous SiO_2_·nH_2_O, which is then deposited mainly within cell walls and on their external surfaces, forming a layer that strengthens the structure of plant tissues [51,54,55,56,57,58,59,60,61]. This phenomenon plays an important role in increasing the stiffness and mechanical strength of plants, as well as in limiting water loss [51,54,55,56,57,58,59,60,61].

Numerous studies have shown that silicon accumulation in plants can enhance their resistance to abiotic and biotic stresses, potentially contributing to improved crop yields and ecosystem stability [62,63,64,65].

Silicon improves plant resistance to drought, salinity, high temperatures, and heavy metal toxicity, and also limits the development of pathogens and pests by creating a physical barrier and inducing defense mechanisms [62,63,64,65,66]. In agricultural plant–soil systems, silicon fertilizers are widely used to increase the productivity of silicon-accumulating crops such as rice, corn, wheat, or sugarcane, especially in (sub)tropical regions where soils are often characterized by a low content of available forms of silicon [62,63,64,67]. The role of silicon in improving crop quality and nutritional value is also increasingly emphasized [62,63,64]. The main plant sources of silica include rice husks and straw, sugarcane bagasse and leaves, corn cobs and ears, wheat, and various kinds of fruit waste [65,68,69,70,71,72,73]. These materials, often treated as waste, constitute valuable and readily available feedstocks for biosilica synthesis, especially in the context of managing agricultural waste and reducing its negative environmental impact [65,69,70,71,72,73]. Their use is consistent with sustainable development strategies and the circular economy [67,69,72,73].

In recent years, interest in producing silica nanoparticles from agricultural waste and by-products has increased [31,35,74,75]. This trend is primarily driven by the availability of these residues and their potential as inexpensive, renewable feedstocks for the production of functional materials [33,74,75].

Nanosilica obtained from biomass often exhibits favorable properties, such as a high specific surface area, a porous structure, and the potential for surface functionalization, making it attractive for applications in catalysis, adsorption, medicine, and materials engineering [31,75,76,77,78]. Further development of research in this area is focused on optimizing synthesis methods, improving control over particle size and morphology, and increasing the efficiency and scalability of production processes [31,35,75].

## 3. Agricultural Wastes Rich in Silica as Feedstocks for the Synthesis of Silica Materials

Based on the analysis of available literature data, substantial differences can be observed in the silica content of ashes obtained from different plant-derived materials [1,38,74,75].

These differences result primarily from the chemical composition of the biomass, including its mineral content and the intrinsic ability of a given plant species to take up and accumulate silicon [1,38]. They are also strongly influenced by plant growth conditions, such as soil type, climate, water availability, and fertilization regime [36]. In addition, the processing parameters used for silica recovery, including combustion temperature, exposure time, oxygen availability, and post-treatment procedures, play a crucial role in determining the purity, morphology, porosity, and degree of crystallinity of the resulting silica [1,32,75,79].

Materials such as rice husk, sugarcane bagasse, wheat straw, and corn residues are characterized by relatively high silica contents, which makes them attractive feedstocks for the synthesis of biosilica and functional materials with high added value [1,70,80,81,82,83,84]. Other agro-industrial residues, including cassava tuber peels and palm kernel shells, have also been considered as alternative sources of silica-rich ash or silica-containing materials [74,75]. Although their silica content may be lower or more variable than that of rice husk, their local availability, low cost, and current underutilization make them relevant from the perspective of circular bioeconomy and waste valorization [9,32,74,75].

Particular attention should also be given to naturally silicon-accumulating plants. Horsetail (*Equisetum arvense* L.) is recognized as one of the richest botanical sources of biogenic silica, while stinging nettle (*Urtica dioica* L.) may also accumulate amorphous silica in plant tissues. These species are especially interesting as potential local biomass sources for future studies focused on the extraction of biogenic silica and its transformation into functional materials for environmental applications.

Other sources, such as fruit wastes or biomass from plants with a lower capacity for silicon accumulation, for example, potato residues, generally contain much smaller amounts of silica. Nevertheless, their wide availability, continuous generation, and low acquisition costs make them interesting from the perspective of sustainable development. The inclusion of such feedstocks in technological processes may contribute to improving biomass utilization efficiency and reducing the environmental burden associated with agricultural and agro-industrial waste, even if they require more advanced methods of silica concentration or purification.

The selection of plant materials discussed in this manuscript, such as potato and maize, reflects not only their relevance in the literature but also their practical importance under Polish agroclimatic conditions. Potato and maize are widely cultivated in Poland and generate substantial amounts of agricultural or processing residues, while nettle and horsetail are commonly occurring silicon-containing plants. Therefore, their inclusion reflects not only literature-based relevance, but also the planned direction of future experimental work. In subsequent studies, locally available biomass from these plant sources will be considered for biosilica extraction, functionalization, and application in environmental remediation.

### 3.1. Rice Husk and Rice Straw

Rice husk, a by-product of rice milling, represents approximately 20% of the grain mass and is widely available, particularly in rice-producing regions of Asia [85,86,87]. Its lignocellulosic structure and substantial silicon content make it a promising feedstock for biosilica production [81,85,86,87,88].

After the combustion process, the resulting ash, referred to as rice husk ash (RHA), contains a very high amount of silica, reaching as much as 93.20%. Depending on the combustion conditions (temperature, oxygen access, process duration), this silica may occur mainly in an amorphous or partially crystalline form, which is of key importance for its further use in the synthesis of materials with specific properties. An appropriately controlled combustion process makes it possible to obtain ash of high purity and a large specific surface area, which makes it a valuable precursor for the production of nanosilica, adsorption materials, or additives for cements and composites [80,81,85,86,87,88,89,90,91]. Rice straw is another lignocellulosic residue with considerable potential for biosilica recovery. Its ash may contain up to 75% SiO_2_, making it a promising alternative silica source [82]. However, its complex composition and low bulk density may complicate processing and transportation. Controlled conversion of rice straw into silica-rich materials offers an alternative to open-field burning and enables its utilization in the production of adsorbents and other functional materials [80,82,92,93].

### 3.2. Corn

Corn cobs are widely available lignocellulosic residues that can serve as precursors for silica recovery [70,80]. Their ash may contain approximately 50–60% SiO_2_, together with minor amounts of calcium, potassium, and magnesium oxides. The mineral composition depends on cultivation conditions and combustion parameters, which influence the purity and properties of the recovered silica. These characteristics support the potential use of corn-cob-derived materials in adsorption and other environmental applications [94,95,96,97,98]. Ash derived from corn ears may contain approximately 64% SiO_2_ and can exhibit relatively uniform particle size, potentially reducing additional grinding requirements [66,69]. The recovered silica may possess a developed porous structure and a high specific surface area, making it suitable for adsorption, catalysis, and composite applications [91,92,93,94,95].

### 3.3. Sugarcane

Sugarcane processing generates substantial quantities of bagasse, straw, and leaf residues [99,100,101]. Approximately 250–270 kg of bagasse and 200 kg of straw and plant tops may be produced per tonne of processed sugarcane [98]. Although these residues are commonly used for energy generation, their mineral fractions also offer opportunities for silica recovery. The ash yield and composition depend on biomass characteristics and processing conditions [99,100,101]. In addition, leaf residues are often burned in fields in order to quickly prepare the soil for the next production cycle, which contributes to serious air pollution problems, including the emission of particulate matter, nitrogen oxides, and greenhouse gases.

Unused bagasse left in the environment may also promote the proliferation of insects and microorganisms that pose potential health risks.

Its high moisture and organic matter contents favor fermentation processes, further complicating storage and transport. About 50% of the generated residues are used in distilleries and sugar plants as an energy source, mainly through their combustion for the generation of process steam and electricity. Such use is in line with the concept of energy self-sufficiency of industrial plants. The remaining part of the biomass is, however, often stored or undergoes uncontrolled burning, which leads to the formation of ash in an amount of about 1–4% of the initial biomass mass. Although this amount may seem small, on an industrial scale it means significant quantities of material that require further management [99,100,101].

Ash obtained from bagasse and sugarcane leaves is characterized by a high silica content, amounting to about 81.6% and 80.14%, respectively, which makes these materials an attractive feedstock for biosilica synthesis. Depending on combustion conditions, this silica may assume an amorphous or partially crystalline form, which affects its physicochemical properties and potential applications [99,101,102]. The high SiO_2_ content, combined with the high availability of the raw material and the low cost of its acquisition, makes sugarcane waste a promising alternative to traditional silica sources [1,83,101,102,103,104].

Sugarcane-derived silica can be further processed into nanosilica, porous adsorbents, composites, and catalyst-related materials, supporting the valorization of these agricultural residues [105,106].

### 3.4. Fruits

Fruit-processing residues, including coconut and walnut shells, orange and banana peels, and selected coffee wastes, have been investigated as alternative feedstocks for silica-containing materials [21,72,73,107,108,109,110,111,112,113,114,115,116]. Although their silicon content is generally lower and more variable than that of major silica-accumulating agricultural residues, their availability and lignocellulosic composition make them potential secondary feedstocks [72,73,108,111,113,114,115]. Thermal or chemical processing may facilitate silica recovery and influence the porosity and surface properties of the resulting materials [108,111,113,114,115,116]. Banana peels have been reported to contain approximately 0.35% silica, substantially less than silica-rich residues such as rice husk ash [72,117,118,119,120]. Their potential utilization therefore depends on feedstock availability and the efficiency of silica recovery, while organic components may influence material properties during thermal processing [108,117,118,119,120]. Coconut and walnut shells, as well as coffee-processing residues, have also been investigated as precursors for silica-containing and hybrid materials [110,111,112,113,121,122,123,124,125,126]. However, their suitability for biosilica production requires consideration of silicon content, mineral impurities, and the processing conditions needed to recover silica of sufficient purity.

### 3.5. Potatoes

Potato plants are generally considered low silicon accumulators compared with crops such as rice and sugarcane [68,127,128]. Silicon uptake depends on the availability of soluble silicon species, transporter activity, and cultivation conditions [68,127,128,129]. Soil-related differences in the uptake and distribution of other mineral elements, including Ca, K, S, and Fe, have also been reported in potato plants [130]. Puppe et al. [68] reported relatively low silicon contents in potato leaves (up to 0.08% of dry mass) and roots (up to 0.30% of dry mass), whereas silicon concentrations in tuber skin and flesh were below the detection limit of the analytical method used. These findings indicate that potato tubers are not a particularly promising source of silicon for biosilica production.

Although silicon may contribute to stress tolerance in potato plants [129,131], their low silicon accumulation limits their suitability as primary feedstocks for biosilica production. Selected potato residues may nevertheless be considered supplementary sources when local availability and processing feasibility justify their use [68,127,128,129,130,131].

### 3.6. Wheat

Wheat-processing residues, including straw, chaff, and husks, are widely available lignocellulosic materials that can serve as precursors for silica recovery [83,84,132]. Their cellulose, hemicellulose, and lignin contents influence thermal processing and the properties of the resulting ash. Wheat-derived silica-rich materials have been reported to contain up to 86% SiO_2_, indicating their potential for further purification and use in functional material synthesis [84,132]. A summary of the reported silica-related contents in selected plant materials is presented in Table 1. However, it should be noted that the literature reports these values on different analytical bases, including Si content in biomass, SiO_2_ content in dry biomass, and SiO_2_ content in ash. Therefore, the values should not be directly compared without considering the material fraction and analytical basis to which they refer. High SiO_2_ contents reported for rice husk, wheat, sugarcane bagasse, and sugarcane leaves generally refer to the ash fraction, whereas substantially lower values reported for fruit wastes and potato tissues refer to the original or dry plant biomass. Accordingly, Table 1 distinguishes the reported parameter and the basis of each value to enable a more accurate assessment of the potential of different agricultural residues as feedstocks for biosilica recovery.

This comparison highlights the importance of feedstock selection for efficient silica recovery [133,134]. Importantly, silica content alone should not be regarded as a direct predictor of the properties or performance of the resulting biosilica. The chemical and structural characteristics of the feedstock, including its mineral composition and lignocellulosic matrix, determine how the biomass responds to thermal and chemical treatment and consequently influence silica purity, porosity, surface area, and structural form. Thus, agricultural residues with similar silica contents may yield materials with substantially different physicochemical properties, depending on both feedstock composition and processing conditions [38,81,83,84,85,86,87,88,89,90,91,132].

The data summarized in Table 1 indicate that rice husk ash has the highest reported SiO_2_ content (93.2%), followed by wheat residues (86.0%), sugarcane bagasse (81.6%), sugarcane leaves (80.1%), and rice straw (75.0%). Lower values are reported for corn-derived ashes, ranging from 50.0 to 60.0% for corn cobs to 64.0% for corn ears [1,70,80,81,82,83,132]. These differences suggest that rice husk, wheat, and sugarcane residues may be particularly attractive feedstocks for silica recovery, although their practical suitability also depends on processing requirements and the quality of the resulting material.

## 4. Methods of Biosilica Synthesis from Plant Materials

For the synthesis of nanosilica from plant materials, methods such as sol–gel, precipitation, hydrothermal synthesis, and acid leaching are most commonly used. The choice of an appropriate technique depends on the expected properties of the final material, such as particle size, specific surface area, or degree of porosity [30,135,136,137,138].

Regardless of the selected method, feedstock preparation involves washing with distilled water to remove contaminants such as dust, mineral salts, and organic residues, followed by drying to remove moisture. This stage is essential for ensuring process reproducibility and the quality of the final product [135,136,137,138].

From an environmental perspective, the main synthesis routes differ in the type of process burden they generate. Sol–gel and acid-leaching approaches require acids and alkalis and may generate substantial washing effluents, whereas thermal treatment and calcination contribute primarily to energy demand. Hydrothermal synthesis requires prolonged heating under pressure, which may also increase process energy requirements. Consequently, the environmental performance of biosilica production depends not only on the renewable origin of the feedstock but also on reagent consumption, water use, thermal energy demand, and waste management. These factors should be considered when comparing biomass-derived routes with conventional silica production [11,12,13,14,15,16,32,75]. In addition to the principal synthesis routes discussed below, other approaches include alkaline extraction followed by chemical precipitation, microwave-assisted processing, and controlled thermal treatment. These techniques may be applied independently or combined with other processing steps to improve silica recovery and control material properties. Their effectiveness depends on feedstock composition, operating conditions, and energy and reagent requirements [1,30,135,136,137,138]. The drying conditions of plant materials may differ depending on the applied research procedure, as presented in Table 2 [139,140,141]. This variability includes, among other things, drying temperature, process duration, and the method used (e.g., convective drying, vacuum drying, or freeze-drying). These differences may significantly affect the properties of the final material, particularly its reactivity, internal structure, and the efficiency of subsequent synthesis stages. Properly selected drying conditions make it possible to preserve the desired biomass structure and facilitate its later chemical and thermal processing.

### 4.1. Sol–Gel Method

**Figure 2:**
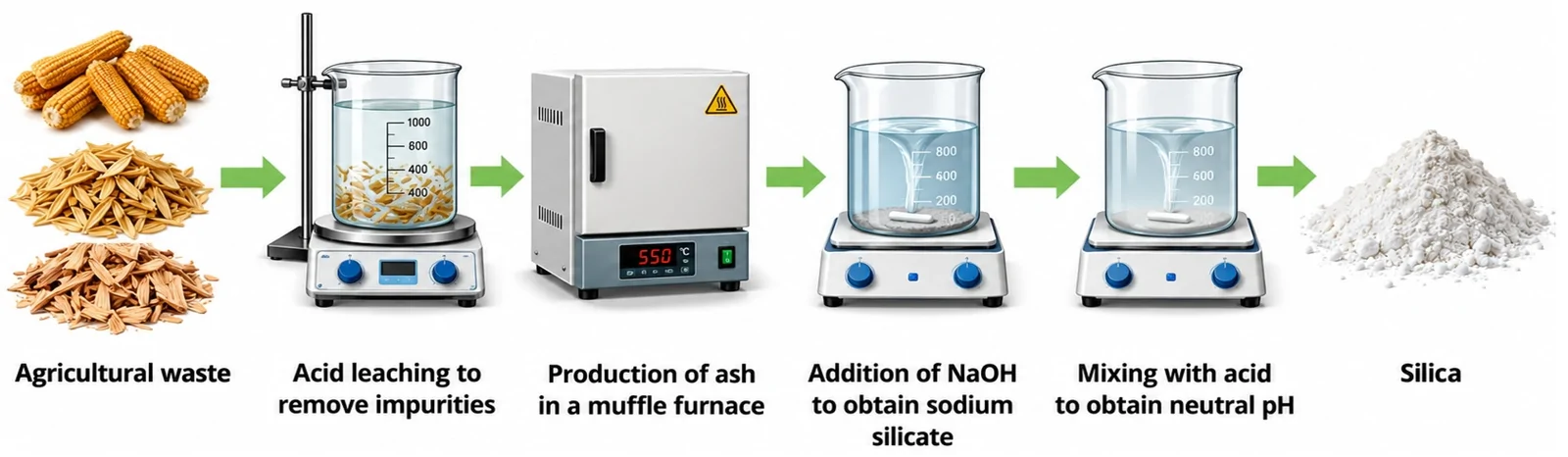
Scheme of the sol–gel method for obtaining silica from agricultural waste (prepared by the authors).

The sol–gel method (Figure 2) is one of the most commonly used approaches for obtaining silica from plant materials [1,30,145,148]. This process involves the transformation of a colloidal system (sol) into a three-dimensional gel network through hydrolysis and condensation reactions, resulting in the formation of Si-O-Si bonds. The obtained material is characterized by high uniformity, high purity, and the possibility of precise control of physicochemical properties such as porosity or particle size [148,149,150,151,152]. Thanks to this, silica obtained by the sol–gel method can be further processed into various forms such as powders, fibers, or thin films, which significantly broadens the range of its applications [1,30,145,148,149,150,151,152,153].

In biomass-derived silica production, sol–gel processing is often preceded by acid leaching as a feedstock-purification step aimed at removing metallic contaminants such as K, Na, Ca, Mg, Al, and Fe. This pretreatment should be distinguished from acid leaching used as the principal silica-recovery route described in Section 4.3. At this stage, partial decomposition of the lignocellulosic matrix also occurs, which increases silica availability and facilitates its further processing [154,155]. The most commonly used reagents include both inorganic acids such as HCl and H_2_SO_4_, and organic acids, including oxalic acid, citric acid, and L-cysteine hydrochloride monohydrate [72,93,135,154,155,156,157,158].

The next stage involves controlled combustion of the material to produce ash, which can subsequently be processed to obtain amorphous silica with high reactivity, small particle size, and a high specific surface area. Combustion process parameters such as temperature and duration are of key importance for obtaining the desired material structure and avoiding the transformation of silica into crystalline forms. The obtained ash is then treated with an alkali solution (most often NaOH), as a result of which silica dissolves and sodium silicate is formed according to the reaction [72,84,159].

SiO_2_ + 2NaOH → Na_2_SiO_3_ + H_2_O

The resulting sodium silicate solution is then acidified, which leads to silica precipitation according to the reaction:

Na_2_SiO_3_ + H_2_SO_4_ → SiO_2_ + Na_2_SO_4_ + H_2_O

The final stage involves washing the precipitate with distilled water to remove residual reagents, followed by drying. Depending on the processing conditions, silica with different structural properties can be obtained, making the sol–gel method a versatile approach for synthesizing nanostructured materials [85].

#### 4.1.1. Influence of Process Parameters in the Sol–Gel Method on the Properties of Nanosilica

The properties of the final product are significantly affected by process parameters, in particular: (i) the type of acid used, (ii) alkali concentration, (iii) combustion temperature and time, and (iv) pH during silica gel formation. Appropriate control of these factors enables precise shaping of the structure, morphology, and surface properties of the obtained silica [135,160,161,162].

##### Type of Acid Used

**Figure 3:**
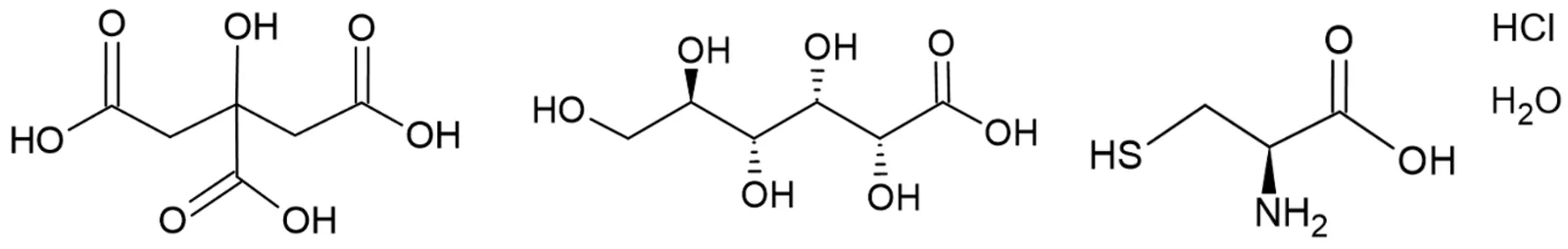
Structural formulas of citric acid, gluconic acid, and L-cysteine hydrochloride monohydrate (prepared by the authors).

In recent years, there has been growing interest in the use of organic acids such as citric acid and L-cysteine hydrochloride monohydrate (Figure 3), which constitute a more environmentally friendly alternative to strong mineral acids. Their use not only allows the costs of wastewater treatment to be reduced, but may also beneficially affect the properties of the obtained silica, including its specific surface area and porosity [101,136,158,163,164]. Citric and oxalic acids also act as effective complexing agents, forming soluble complexes with metal ions and thereby facilitating the removal of inorganic impurities from biomass. This chelation mechanism contributes to the effectiveness of organic acid pretreatment in biosilica production [155,163,165].

Studies have shown that L-cysteine hydrochloride monohydrate may be more effective in removing metallic contaminants than citric acid, which is attributed to the presence of chloride ions increasing extraction efficiency [158]. In addition, gluconic acid exhibits high effectiveness in removing metal oxides such as K_2_O, Na_2_O, and Al_2_O_3_, achieving efficiencies of 92–93%, 89–93%, and 95–97%, respectively [165].

The type of acid used may influence the specific surface area and porosity of the resulting silica through differences in impurity removal and particle formation. During biomass pretreatment, acid-dependent removal of metallic impurities can affect subsequent structural transformations during thermal processing [155,158,165]. During silica precipitation, the acid type and resulting pH conditions may influence particle formation and aggregation, thereby affecting the final textural properties. For example, Thuadaij and Yodyingyong [160] reported a specific surface area of 554.01 m^2^/g for silica derived from sugarcane bagasse ash using citric acid, exceeding the values obtained with sulfuric, oxalic, and acetic acids under the investigated conditions.

##### Alkali Concentration

An important process parameter is also the concentration of the alkali, most often NaOH. An increase in alkali concentration and solvent volume promotes an increase in process yield through more effective silica dissolution and formation of soluble sodium silicate [161,166,167,168]. NaOH is commonly used for silica extraction because it promotes the dissolution of silica and the formation of soluble sodium silicate [161]. However, its effect on the structural properties and particle size of the final material depends on the synthesis conditions. Although more ordered pore structures and larger particles have been reported for NaOH-catalyzed systems compared with NH_4_OH-catalyzed systems, these observations relate to specific mesoporous silica synthesis conditions and should not be generalized to biosilica extraction processes [169].

In practice, concentrations ranging from 0.5 M to 4 M are used, with optimum values often around 2 M. In the case of rice husk, the optimum concentration lies in the range of 1–4 M [166,167,168], whereas for sugarcane bagasse it is 2–4 M. Selecting an appropriate concentration is essential for balancing process efficiency and control over the structural properties of the material [161,166,167,168,169].

##### Influence of Temperature and Combustion Time

The calcination temperature is one of the key factors affecting silica properties [135,170,171]. The optimum temperature range is usually 600–700 °C.

Increasing the calcination temperature promotes the decomposition and removal of organic constituents, thereby increasing the relative silica content. However, the removal of inorganic impurities depends on their chemical nature and may require additional acid-leaching treatment [135,170,171].

**Figure 4:**
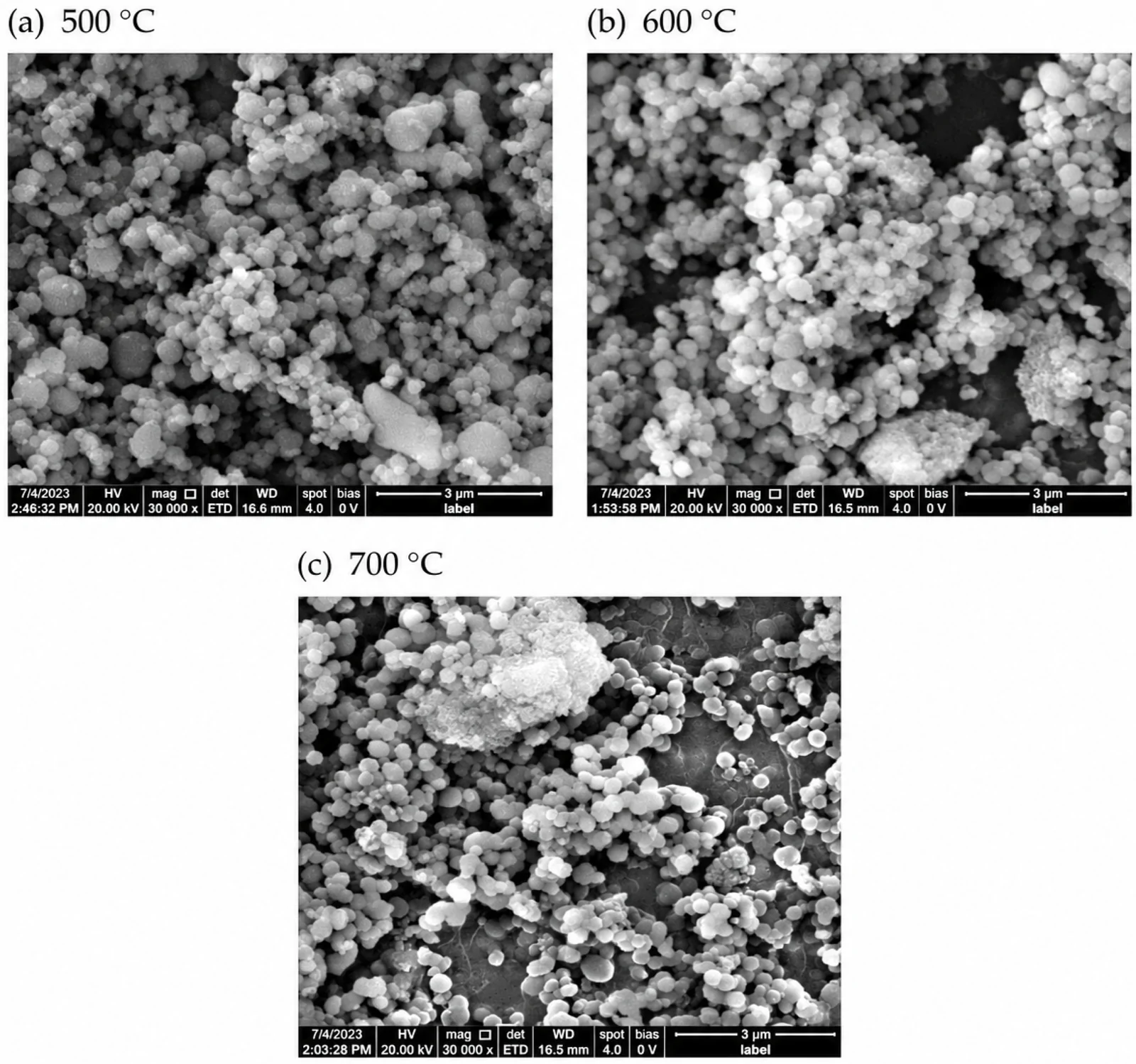
SEM images of silica obtained from corn stalk by calcination at (**a**) 500 °C, (**b**) 600 °C, and (**c**) 700 °C. Reproduced from Ref. [170] with permission from Springer.

**Figure 5:**
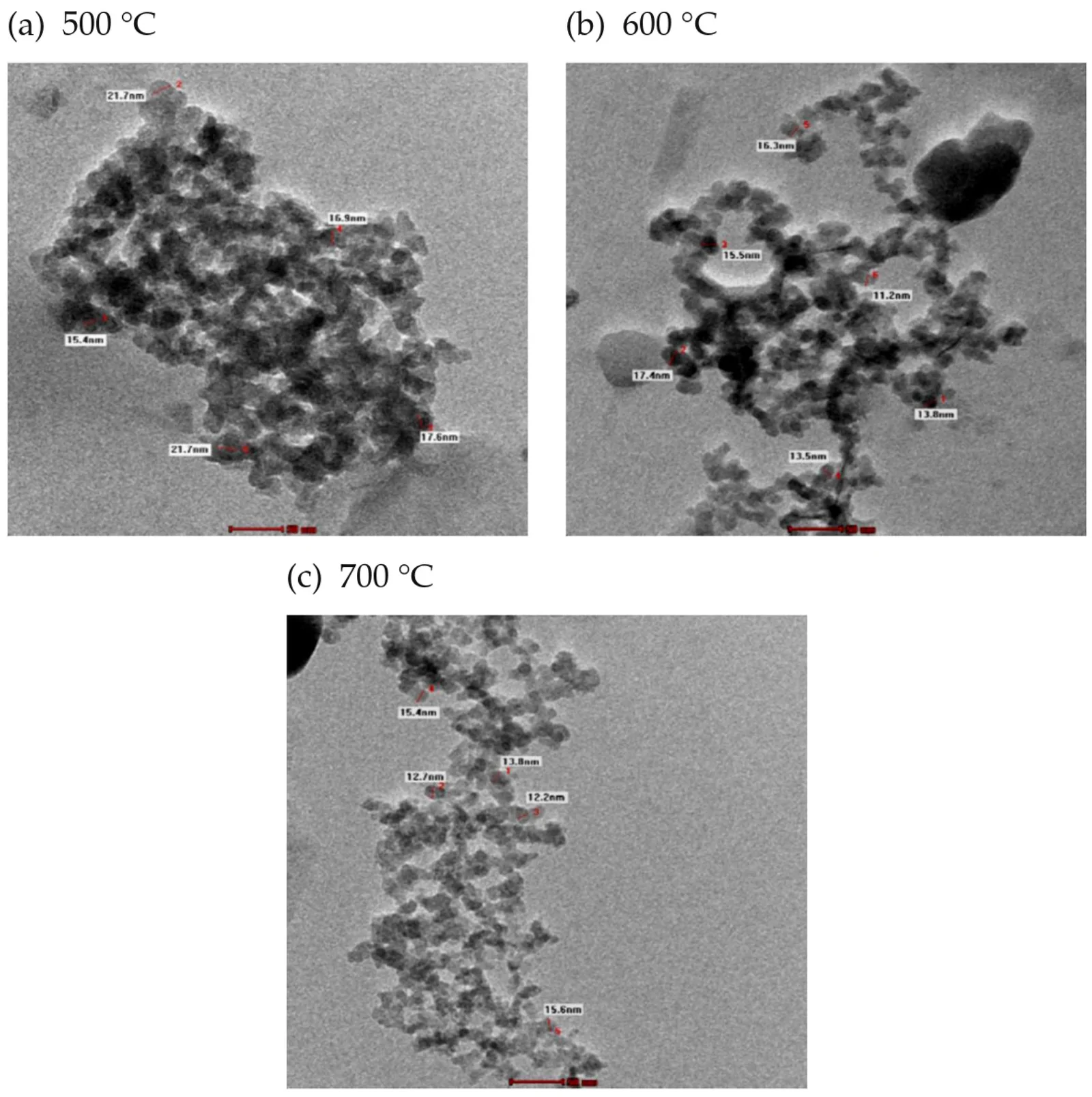
TEM images of silica obtained from corn stalk by calcination at (**a**) 500 °C, (**b**) 600 °C, and (**c**) 700 °C. Reproduced from Ref. [170] with permission from Springer.

For example, silica obtained from corn stalks at 700 °C exhibited higher purity (98.1%) compared with material obtained at 500 °C (91.1%). At the same time, temperature affects the degree of particle agglomeration; materials obtained at higher temperatures exhibit a lower tendency to form aggregates and possess a more homogeneous structure (Figure 4 and Figure 5) [170].

However, excessively high processing temperatures (above 800 °C) may lead to unfavorable structural changes, including decreases in specific surface area and pore volume associated with silica crystallization [172].

**Figure 6:**
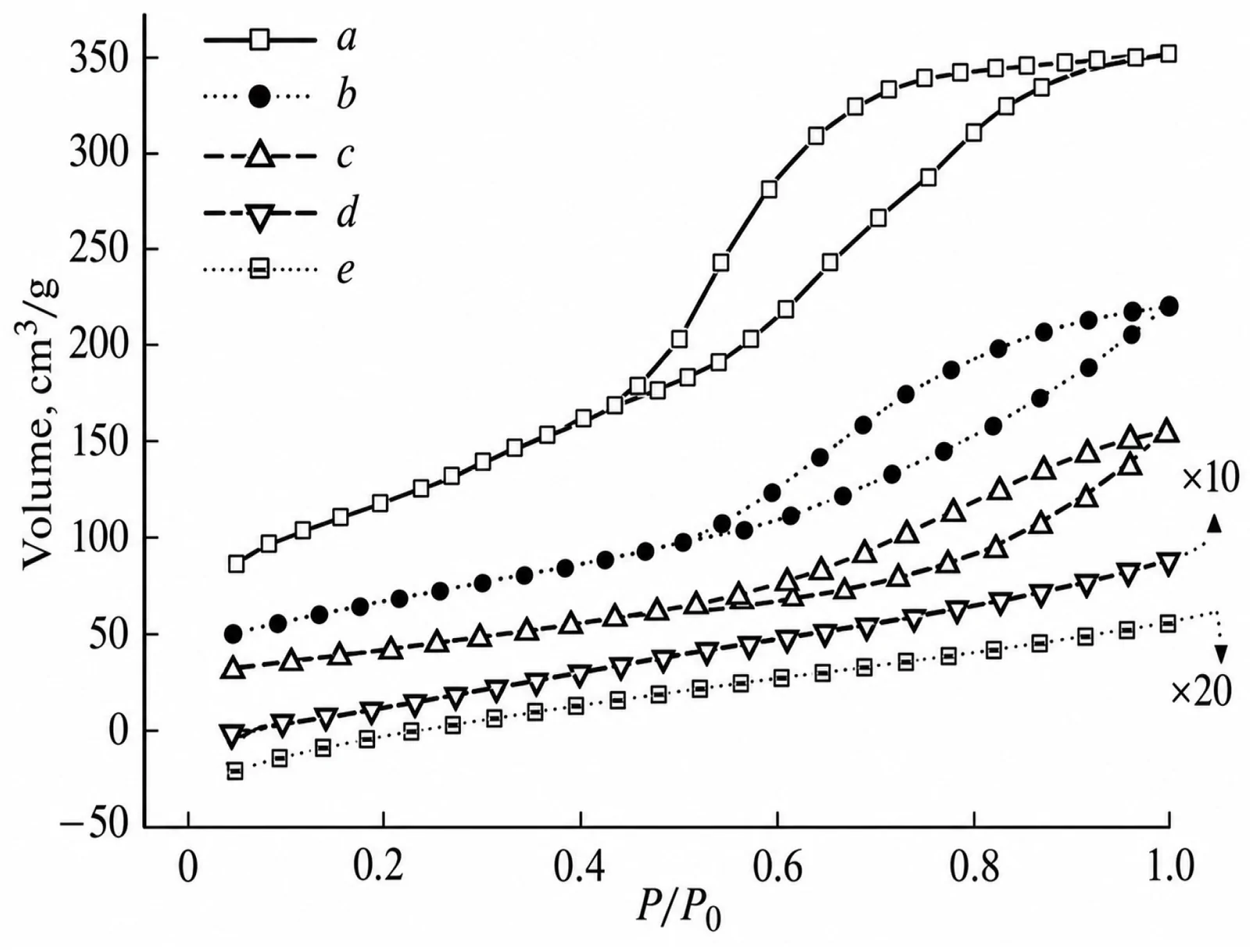
Low-temperature nitrogen adsorption and desorption isotherm curves of the silica sample obtained from oat straw before (a) and after heating at different temperatures: (b) 600 °C, (c) 800 °C, (d) 1000 °C, and (e) 1200 °C. Reproduced from Ref. [173] with permission from Springer.

**Figure 7:**
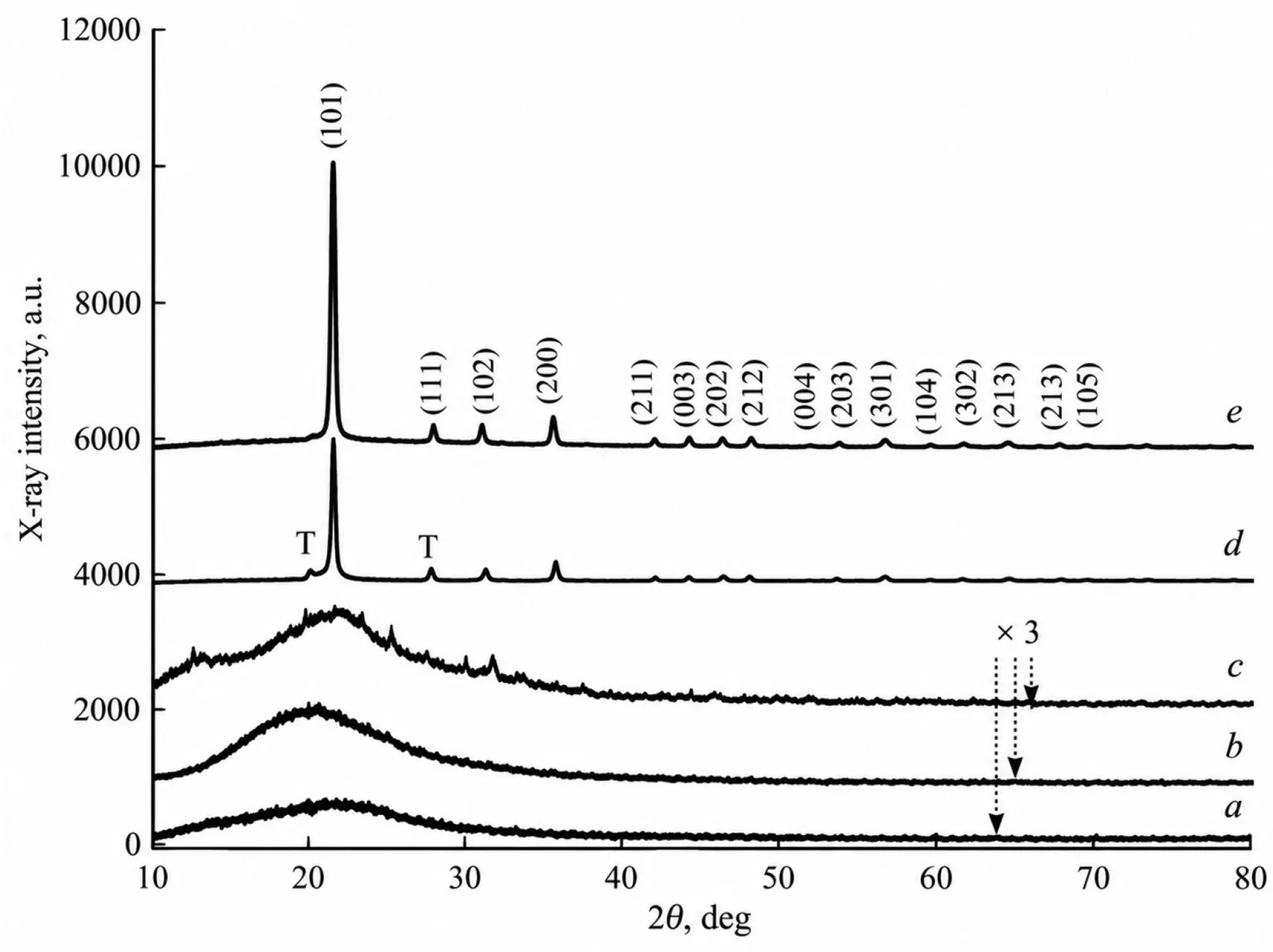
XRD patterns of the silica sample obtained from oat straw before (a) and after heating at different temperatures: (b) 600 °C, (c) 800 °C, (d) 1000 °C, and (e) 1200 °C. Reproduced from Ref. [173] with permission from Springer.

This transformation may be confirmed by X-ray diffraction (XRD) analysis and by measurements of specific surface area using the BET method, where clear changes characteristic of crystalline silica are observed (Figure 6 and Figure 7).

Analysis of the low-temperature nitrogen adsorption–desorption isotherms presented in Figure 6 indicates that, according to the IUPAC classification, most of the studied samples exhibit type IV(a) isotherms with distinct hysteresis loops, characteristic of mesoporous materials. The isotherms of oat-straw-derived samples treated at temperatures below 1000 °C exhibit similar profiles, although the hysteresis loops gradually change with increasing temperature.

After increasing the temperature to 1000 °C, the hysteresis loop disappears and the shape of the isotherm changes toward type II, which is characteristic of non-porous or macroporous materials. This change indicates degradation of the mesoporous structure and a decrease in the developed specific surface area of the material [173].

X-ray diffraction (XRD) analysis presented in Figure 7 indicates that the silica sample obtained from oat straw before thermal treatment exhibits a broad reflection in the range of 2θ = 14–36°, which is characteristic of the amorphous silica structure. After treatment at 600 °C, the first weak reflections corresponding to the crystalline phase appear in the diffraction pattern, overlapping the broad amorphous-phase peak. With an increase in temperature to 800 °C, a clear intensification of the signals originating from the crystalline phase is observed, and they begin to dominate in the diffraction image. At 1000 °C and above, the features characteristic of the amorphous structure disappear, and the XRD pattern exhibits sharp, well-defined reflections corresponding to the crystalline silica. This change indicates a phase transition and structural reorganization of the material as a result of intensive calcination [173,174].

The optimum combustion time is usually 2 h to 4 h, which allows a material with a high specific surface area, developed porosity, and high purity to be obtained. Extending the calcination time leads, in turn, to a decrease in specific surface area and an increase in the degree of silica crystallinity [92,175,176].

##### Effect of pH

Precise pH control during gelation is essential for regulating the physicochemical properties of the resulting silica. This parameter directly affects particle morphology, specific surface area, and particle size [162,174]. With increasing pH value, a decrease in the specific surface area of the material is observed. Studies by Liou et al. showed that for silica obtained from rice husk ash, the highest specific surface area (634 m^2^/g) was obtained at pH = 3, whereas the lowest (237 m^2^/g) was obtained at pH = 9 [174]. Moreover, it was found that at pH values above 8, the formed gels become unstable and undergo partial dissolution, whereas at pH = 11, almost complete degradation of the gel structure is observed in an aqueous environment.

Thus, the optimal gelation pH depends on the desired material properties and process objectives. While acidic conditions may favor the development of a higher specific surface area, near-neutral conditions can be advantageous for silica recovery and gel formation [162,174].

#### 4.1.2. Advantages and Disadvantages of the Sol–Gel Method

Despite its numerous advantages, the sol–gel method has certain limitations, including relatively long processing times and the need to perform some stages at elevated temperatures. Nevertheless, it enables the production of materials with high purity, a homogeneous structure, and a large specific surface area, making it particularly attractive for nanomaterial synthesis [177,178,179].

Compared with the hydrothermal method, the sol–gel process is less energy-intensive and is considered more environmentally friendly, mainly because of reduced pollutant emissions [177].

To optimize biosilica synthesis from agricultural waste, reaction conditions are adjusted by varying the proportions of acids, alkaline solutions, and plant material to improve process yield [177,180].

#### 4.1.3. Examples of Obtaining Biosilica by Sol–Gel Method

For example, Uda et al. [89] added 400 mL of 2.5 M NaOH solution to 20 g of rice straw ash. The mixture was stirred magnetically at 100 °C for 4 h. Subsequently, 2 M H_2_SO_4_ was added to adjust the pH to approximately 7, and the mixture was stirred for an additional 18 h.

**Figure 8:**
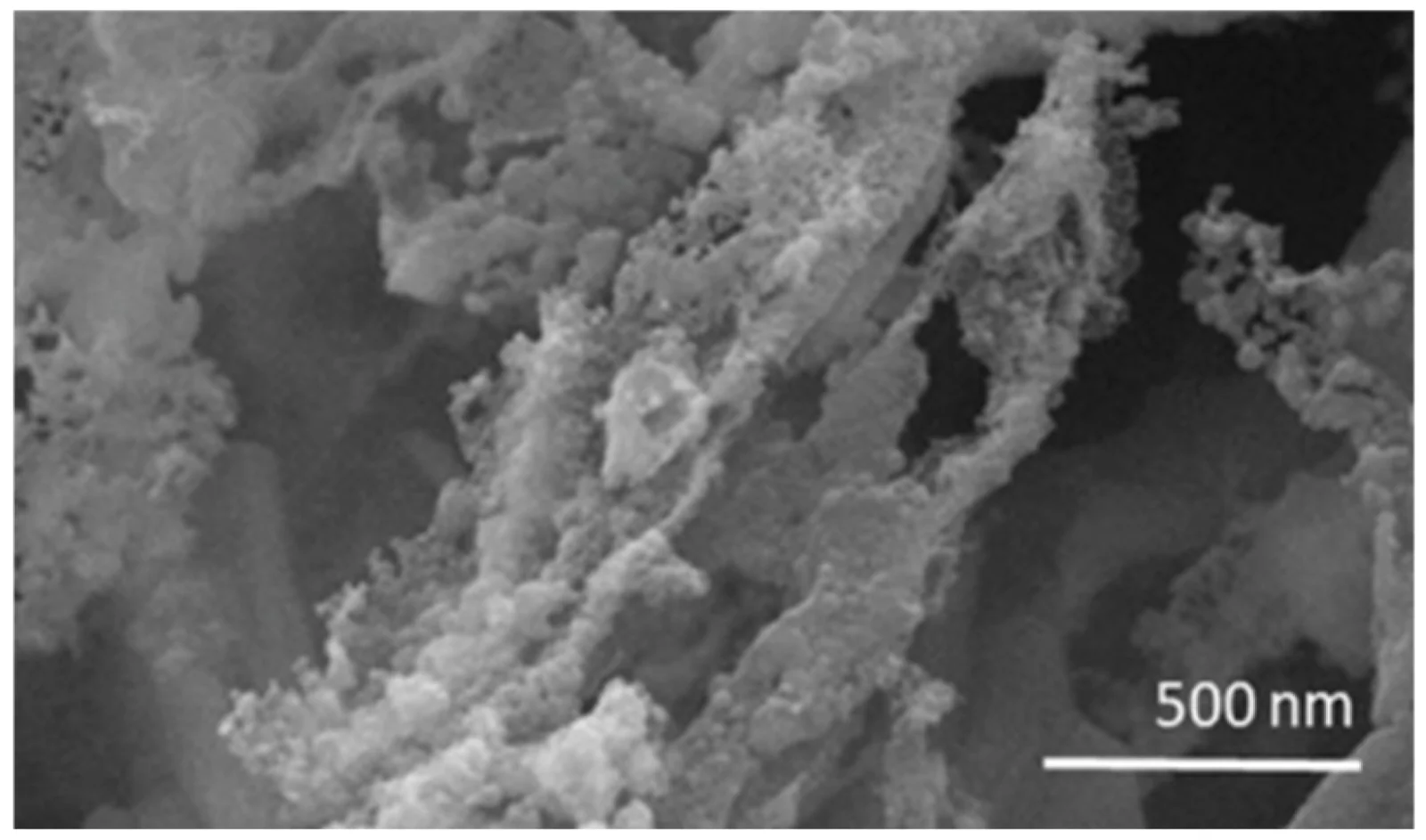
FE-SEM image of rice straw ash. Reproduced from Ref. [89] with permission from Taylor & Francis.

**Figure 9:**
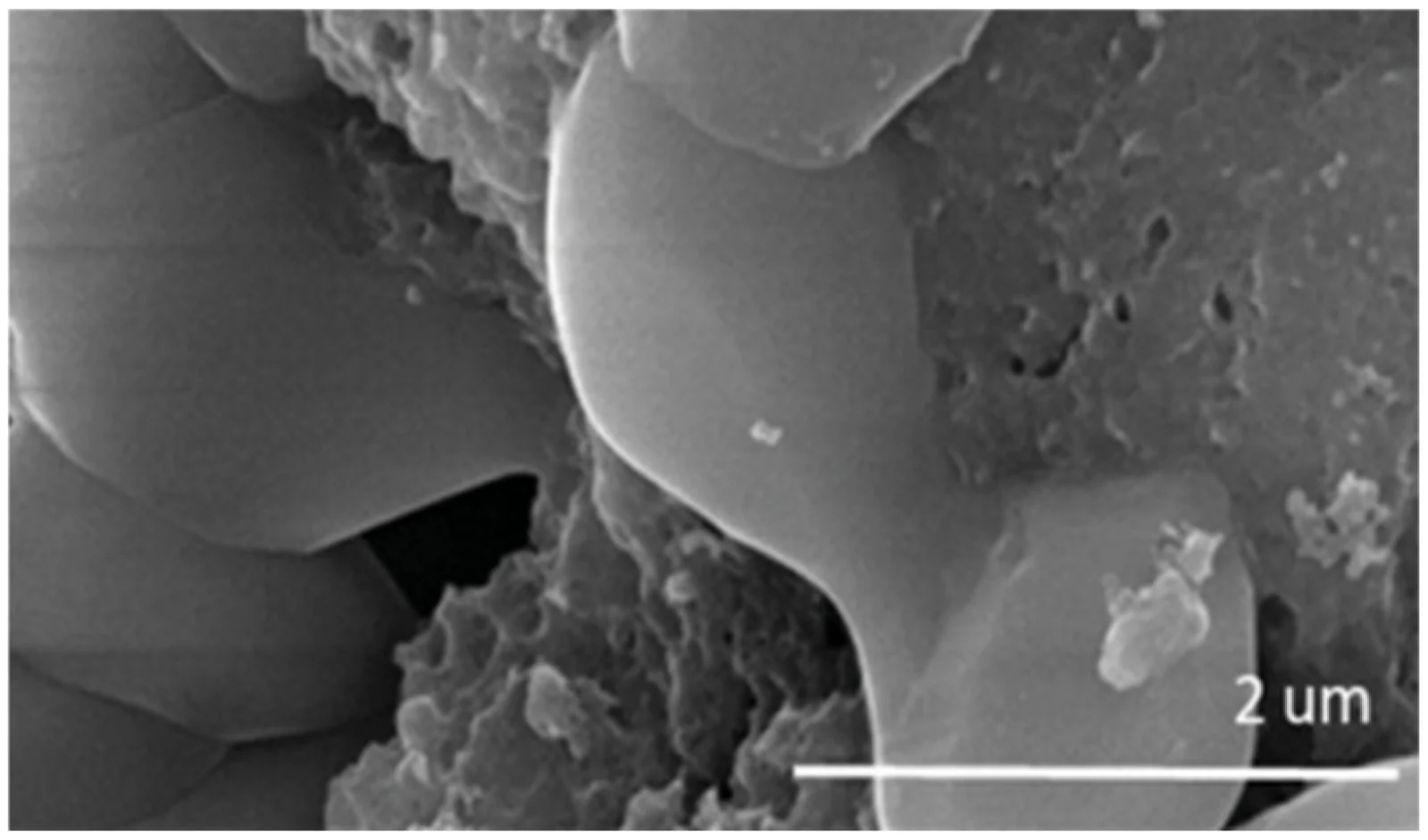
FE-SEM image of synthesized silica from rice straw. Reproduced from Ref. [89] with permission from Taylor & Francis.

The obtained precipitate was washed with ethyl alcohol and distilled water and then dried at 80 °C for 30 min, yielding about 76% silica relative to the initial ash mass. The material obtained was characterized by FE-SEM and compared with ash obtained directly from the combustion of rice straw (Figure 8 and Figure 9) [89].

The FE-SEM image of the ash sample (Figure 8), recorded at a magnification of 150×, shows particles with a granular morphology. After treatment with alkali and acid solutions, silica nanoparticles with significantly more developed porosity were obtained (Figure 9). Studies show that the reaction of silica with NaOH leads to the formation of sodium silicate (Na_2_SiO_3_), with water being formed as a by-product. Then, as a result of acidification of the solution, silica precipitates and inorganic impurities are removed, which favors the formation of a material with high porosity and an amorphous structure [89].

Table 3 summarizes selected conditions used for silica synthesis by the sol–gel method. Considerable variation in process parameters, including ash-production temperature, alkali concentration, and reaction conditions, reflects differences among the feedstocks used. In most cases, NaOH is used as the alkaline agent and HCl as the acidifying reagent, with a final pH close to neutral. This summary shows that despite differences in procedures, it is possible to obtain silica under a wide range of conditions, which confirms the flexibility of the sol–gel method. Recent developments in sol–gel processing have focused on integrating silica recovery with the valorization of other biomass components. Ehlan et al. [181] reported an optimized silica extraction yield exceeding 90% from rice husks, obtaining a mesoporous material with a specific surface area of 279 m^2^/g. Their approach also enabled the production of solid fuel and potassium sulfate fertilizer, highlighting the potential of integrated biorefinery strategies.

### 4.2. Hydrothermal Method

**Figure 10:**
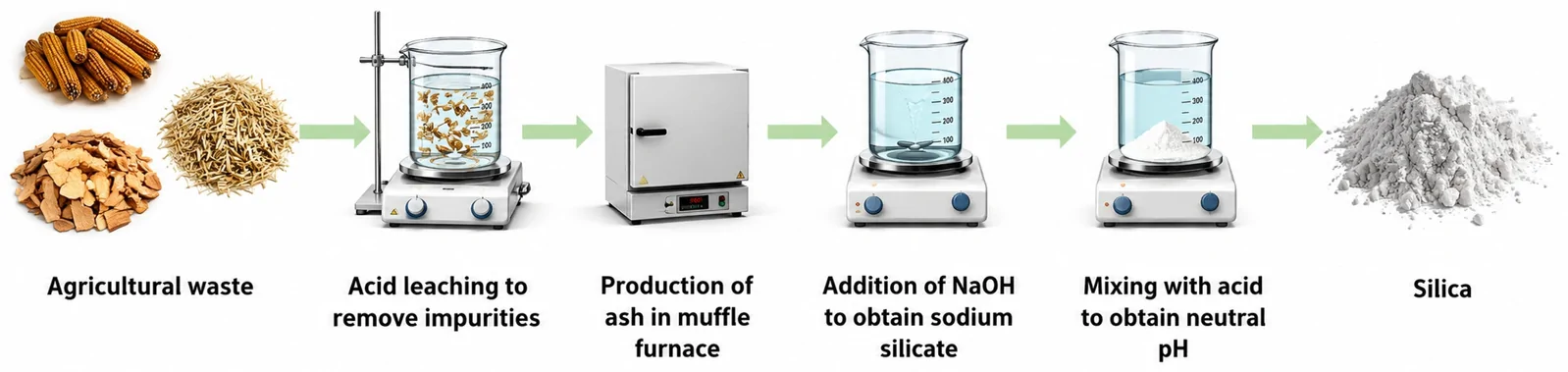
Scheme of the hydrothermal method for obtaining silica from agricultural waste (prepared by the authors).

Hydrothermal synthesis (Figure 10) is a widely used method for obtaining nanomaterials and involves reactions in aqueous media under controlled temperature and pressure conditions [1,189]. Depending on the feedstock and processing conditions, hydrothermal treatment may be conducted at temperatures of approximately 100–260 °C, with the selected temperature influencing the morphology and structural properties of the resulting materials [189,190]. Pressure conditions resulting from the vapor pressure of reaction components play a key role in shaping the properties of the final product [189].

Hydrothermal synthesis enables the preparation of materials with controlled morphology and crystallinity, including crystalline phases that may be difficult to obtain using conventional high-temperature processing [1,190]. The main stages of biosilica synthesis by the hydrothermal method include:

- Mixing and heating—plant material constituting a source of silica is mixed with water and then placed in an autoclave. The system is heated to a specified temperature and maintained under pressure for a defined reaction time [191,192].
- Final treatment—the reaction product may undergo additional purification, including washing or acid leaching with HCl to remove residual impurities and improve the purity of the recovered silica [193].
- Surface modification (capping agents)—additives such as polysaccharides or surfactants may be used to control nanoparticle morphology [194].

Hydrothermal synthesis is carried out in autoclaves, which make it possible to control process parameters such as temperature and pressure. These parameters have a significant influence on particle size, degree of crystallinity, and morphology of the obtained material. 

Bathla et al. [195] reported the hydrothermal synthesis of silica nanowires from rice husk ash. The resulting structures had diameters ranging from 15 to 35 nm and lengths of approximately 0.5 μm, demonstrating the potential of this method to control material morphology.

Hamidu et al. [196] investigated a combined treatment involving acid leaching, hydrothermal processing, and carbonization for silica recovery from rice husks. Treatment with 5% HCl, followed by hydrothermal processing and carbonization, yielded silica with a purity of 98.1% and a recovery yield of 19.8%. This study demonstrates the potential of integrating hydrothermal pretreatment with complementary purification steps.

### 4.3. Acid Leaching Method

**Figure 11:**
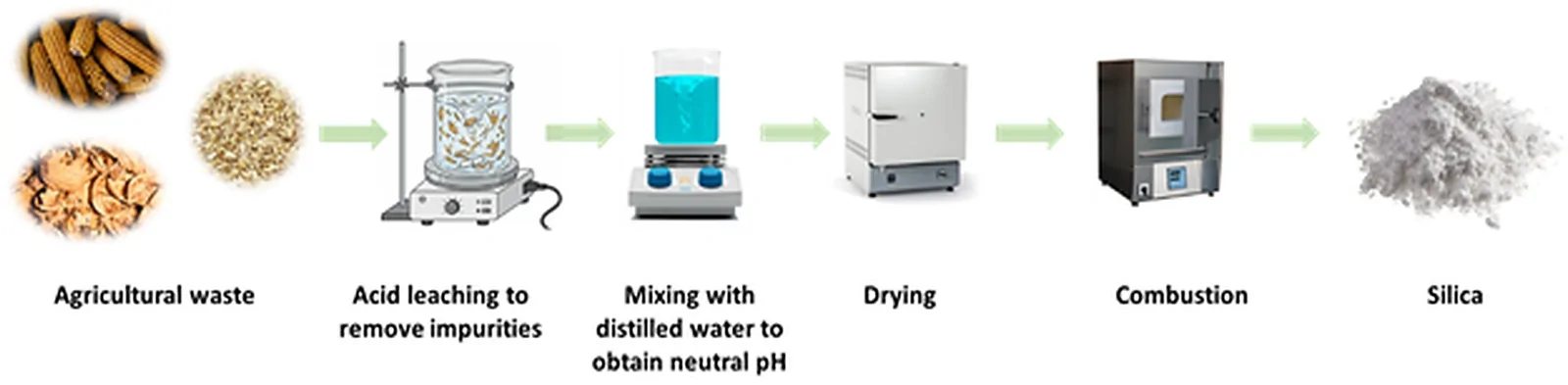
Scheme of the acid leaching method for obtaining silica from agricultural waste (prepared by the authors).

The acid leaching method (Figure 11) involves the selective removal of impurities from solid materials using an acid solution. During this process, acid treatment removes contaminants from plant biomass, increasing the accessibility of silica for subsequent recovery [1,197,198]. In contrast to the sol–gel pathway, where acid treatment may serve as a preliminary purification step before alkaline extraction and gel formation, the acid-leaching route discussed here relies primarily on selective removal of mineral and metallic impurities followed by thermal recovery of silica-rich material. Thus, although the two routes may share individual processing steps, they differ in the dominant mechanism used for silica recovery and final material formation.

The key parameters affecting process efficiency include the choice of solvent (acid), reaction time, and temperature [1].

#### 4.3.1. The Role of Acid Type and Concentration in the Acid Leaching Method

Both inorganic acids, such as nitric, sulfuric, and hydrochloric acids, and organic acids, including citric acid, are commonly used for acid leaching [1,135].

**Figure 12:**
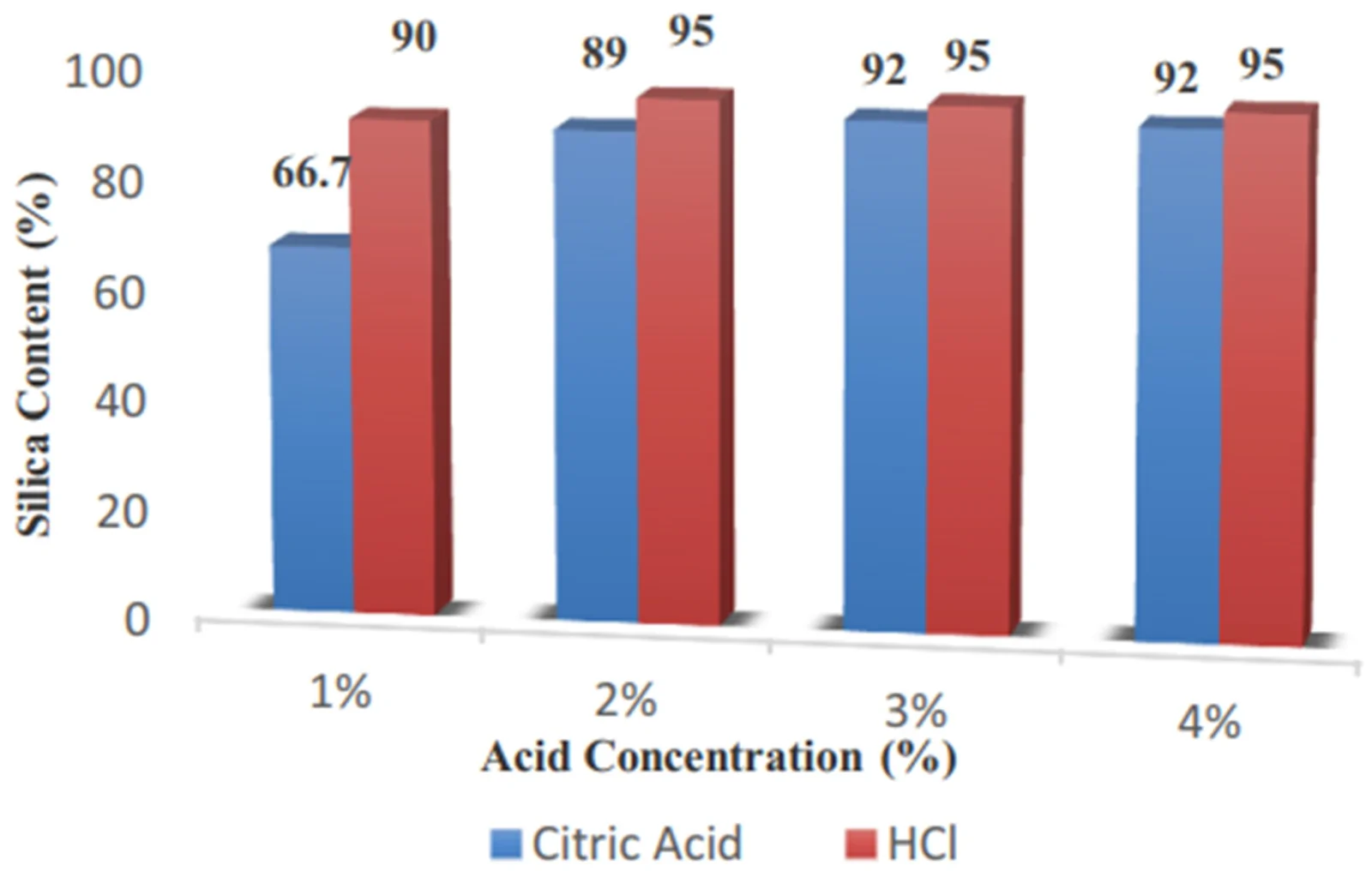
Comparison chart of the amount of silica obtained from palm ash using HCl and citric acid. Reproduced from Ref. [200] under the terms of the Creative Commons Attribution 4.0 License.

Mineral acids are effective in removing metallic impurities, which can facilitate the production of silica with a high specific surface area and a well-developed mesoporous structure [176,199,200]. Organic acids can also be used to obtain silica with favorable textural properties [197,201]. For example, Tessema et al. [176] obtained silica from teff straw with a specific surface area of 739 m^2^/g using HCl, whereas Cahyani et al. [201] reported a specific surface area of 155 m^2^/g for silica derived from rice husk using acetic acid. However, these values cannot be directly compared to assess acid effectiveness because the studies involved different biomass feedstocks and processing conditions. Organic acids may offer advantages in terms of lower corrosiveness, although their environmental performance depends on the specific process conditions [197,201]. Comparisons conducted under identical reaction conditions indicate that inorganic acids can provide higher silica recovery yields than organic acids in certain systems (Figure 12) [200]. The influence of acid type is related not only to its strength but also to its ability to interact with metal-containing impurities present in the biomass. Strong mineral acids promote proton-assisted dissolution of inorganic species, whereas organic acids may additionally remove metal ions through complexation, depending on their dissociation and ligand structure. These differences affect the extent of matrix purification and the accessibility of the silica surface, including surface silanol groups and pore openings. Consequently, acid selection may influence the surface chemistry and adsorption-related properties of the resulting biosilica [136,155,158,165,197,200,201]. Díaz-Tovar et al. [155] investigated the influence of acid type and pH on impurity removal from rice husks and the properties of the resulting biosilica. Under optimized leaching conditions, the obtained material exhibited a purity of 99.45 ± 0.04% and a specific surface area of 318 ± 10 m^2^/g, demonstrating the importance of controlling pretreatment parameters.

#### 4.3.2. Influence of Temperature and Reaction Time on the Acid Leaching Method

The temperature of the leaching process plays an important role in component solubility and silica extraction efficiency.

The leaching temperature is typically in the range of 70–100 °C, with higher temperatures generally accelerating impurity removal. During subsequent calcination, temperatures of approximately 600–700 °C are commonly used to obtain high-purity silica while limiting undesirable structural transformations. Reaction time also significantly affects the properties of the final product.

Leaching times of 60–150 min are commonly reported, although the optimal duration depends on the feedstock and reaction conditions. Extending the treatment may improve impurity removal, but does not necessarily result in a further increase in silica purity or specific surface area [1,155,202,203,204,205].

#### 4.3.3. Examples of Obtaining Biosilica by the Acid Leaching Method

The acid leaching method is relatively simple and economical, and makes it possible to obtain silica of high purity. However, its main disadvantage is the need to use considerable amounts of acids and a relatively long reaction time, which may generate an environmental burden [138,156,178]. For example, Maseko et al. used 50 g of sugarcane biomass, which was first washed with 1200 mL of distilled water and stirred for 2 h, and then dried at 100 °C overnight. The material was then subjected to leaching using 7 wt.% citric acid and sulfuric acid. After the leaching process, the material was washed again with distilled water and dried at 50 °C for 24 h. The next stage involved multistage calcination: 30 min at 310 °C, 60 min at 450 °C, 210 min at 560 °C, and 30 min at 600 °C. As a result of this process, a material containing about 99.7% silica, with a specific surface area of 323 m^2^/g and a pore diameter of about 5 nm, was obtained [138]. 

**Figure 13:**
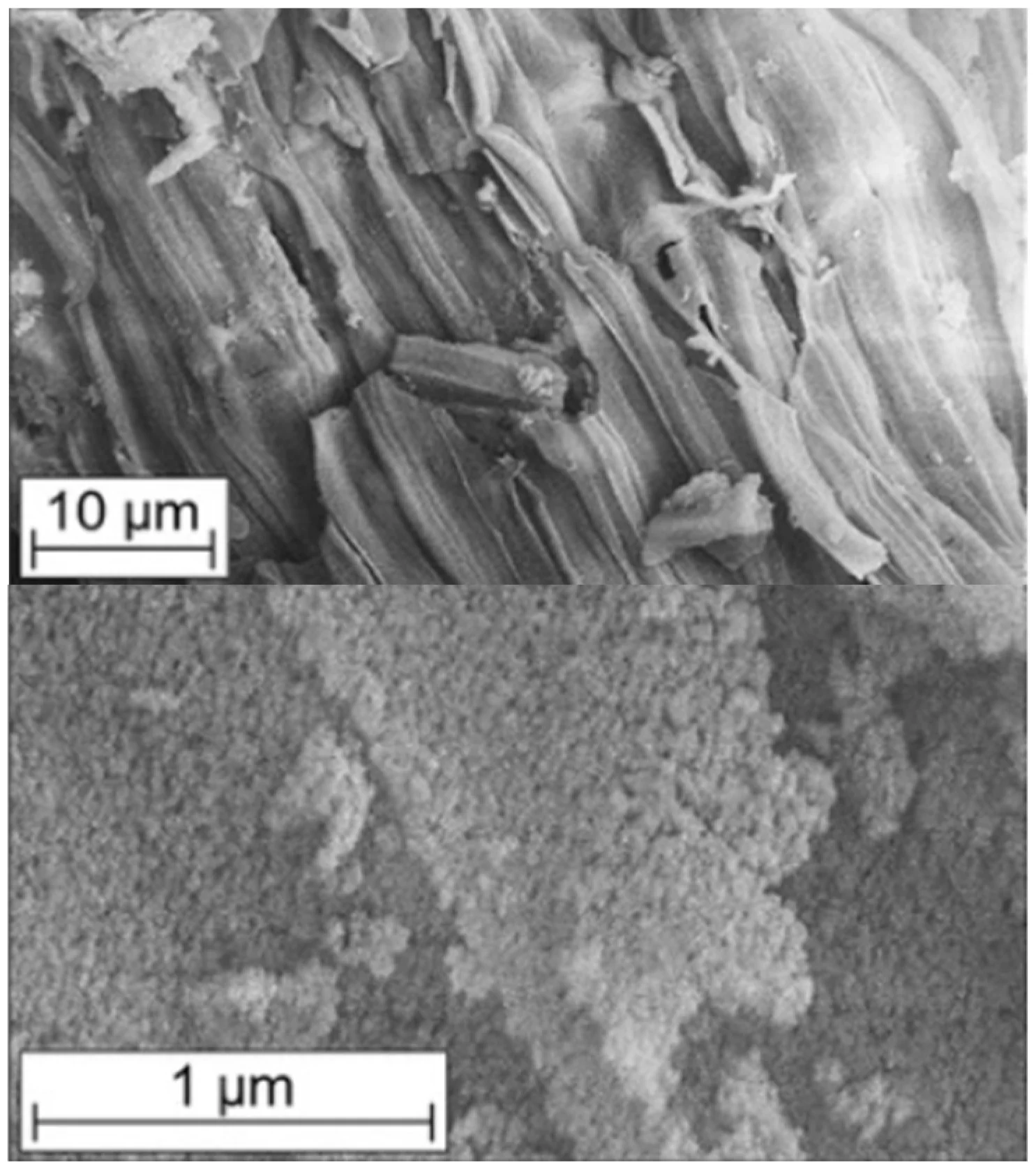
SEM images of rice straw (**top**) and the obtained biosilica (**bottom**). Reproduced from Ref. [156] with permission from Springer.

**Figure 14:**
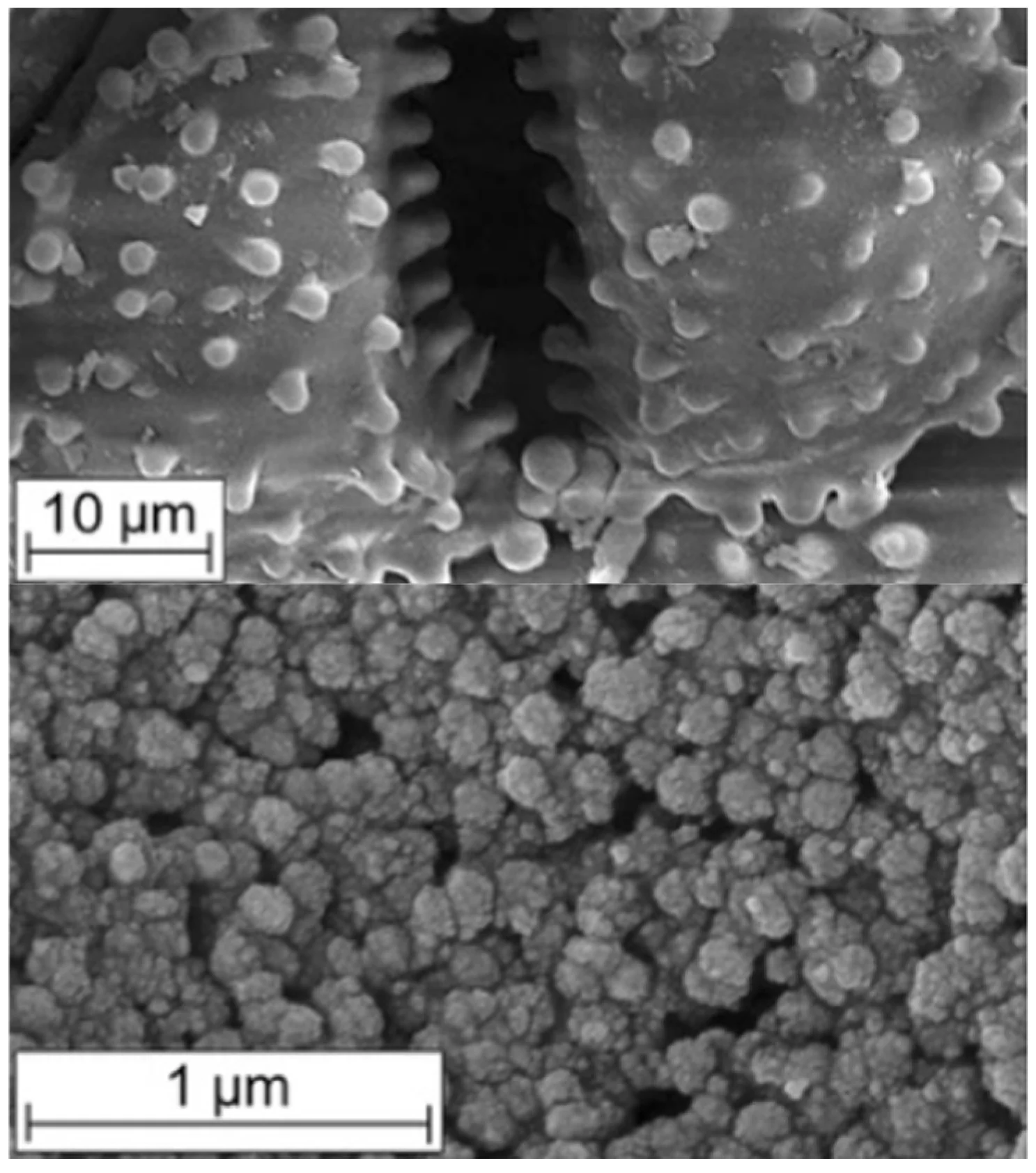
SEM images of horsetail (**top**) and the obtained biosilica (**bottom**). Reproduced from Ref. [156] with permission from Springer.

In another study, Schneider et al. [156] used 100 g of rice straw or horsetail as starting material, together with 1300 mL of distilled water and citric acid. As a result of the synthesis, silica with a purity of 99.7%, a specific surface area of 303 m^2^/g, and pores with a diameter of 2–30 nm was obtained [156]. SEM analysis (Figure 13 and Figure 14) showed that the obtained biosilica is characterized by the presence of empty interparticle spaces associated with particle agglomeration and the development of a porous structure [156].

Table 4 presents an overview of selected acid leaching conditions used for obtaining silica from plant materials such as rice husks, sugarcane bagasse, or oil palm waste. The comparison includes key stages of the process, including acid digestion, washing to a specified pH, drying, and combustion, which determine the purity and properties of the obtained material. The variation in acid type, concentration, and processing parameters highlights the need to optimize treatment conditions for each biomass feedstock and the desired properties of the resulting silica.

## 5. Comparison of Biosilica Production Methods

Direct comparison of biosilica production routes is complicated by differences in feedstock composition, pretreatment, process definition, and reporting basis [115,206,207]. In particular, acid leaching may function either as a preliminary purification step within a sol–gel process or as the principal treatment preceding thermal silica recovery. Therefore, the methods should be compared according to their dominant silica-recovery mechanism rather than solely on the presence of individual processing steps. The sol–gel route offers high flexibility in controlling structure and surface properties, hydrothermal synthesis enables control of morphology and crystallinity under pressurized aqueous conditions, whereas acid leaching provides efficient removal of mineral impurities and may yield high-purity silica. These differences should be considered together with reagent consumption, water demand, and thermal requirements [155,208]. Table 5 therefore provides a comparative assessment of representative synthesis routes in terms of silica yield, purity, specific surface area, reagent consumption, water use, temperature requirements, and major environmental limitations.

From an economic perspective, the main cost-related factors include feedstock collection and pretreatment, reagent consumption, water use, energy demand, processing time, and waste management. Although Table 5 provides a qualitative comparison of these requirements, reliable quantitative production-cost estimates cannot be derived from the available literature due to differences in processing scale, experimental conditions, and reporting practices. Therefore, comprehensive techno-economic assessments are needed to evaluate the commercial feasibility of individual biosilica production routes.

The comparison is based on the quantitative parameters reported in the original studies, without normalizing values obtained from different feedstocks or experimental conditions. Missing data are explicitly indicated as “n.r.” (not reported), and the resulting limitations are considered when interpreting differences between synthesis routes.

Overall, compared with conventionally produced silica, biosilica offers potential advantages associated with the use of low-cost renewable feedstocks, reduced dependence on mineral resources, and waste valorization. However, conventional synthesis routes generally allow more consistent control over silica purity, particle morphology, and structural reproducibility. In terms of adsorption performance and stability, no universal superiority of either material can be assumed, because these properties depend strongly on synthesis conditions, surface chemistry, functionalization, and the target contaminant. Similarly, potential reductions in production cost, energy consumption, and environmental burden are process-dependent and should be verified by comparative techno-economic and life-cycle assessments.

The data presented in Table 5 reveal considerable differences in the physicochemical properties of biosilica obtained from agricultural residues. Sol–gel processing of teff straw yielded silica with a specific surface area of 807.1 m^2^/g [206], whereas microwave-assisted processing of rice husk produced materials with values ranging from 400 to 700 m^2^/g [209]. Acid-leaching treatments resulted in specific surface areas of 234.25 m^2^/g for rice husk [136], 303 m^2^/g for rice straw and horsetail [156], and 323 m^2^/g for sugarcane biomass [138]. Silica purities of 99.7% were reported for the latter two materials. However, differences in processing conditions and incomplete reporting of silica yield and purity limit direct comparisons between these studies.

### 5.1. Influence of Temperature on Silica Structure

**Figure 15:**
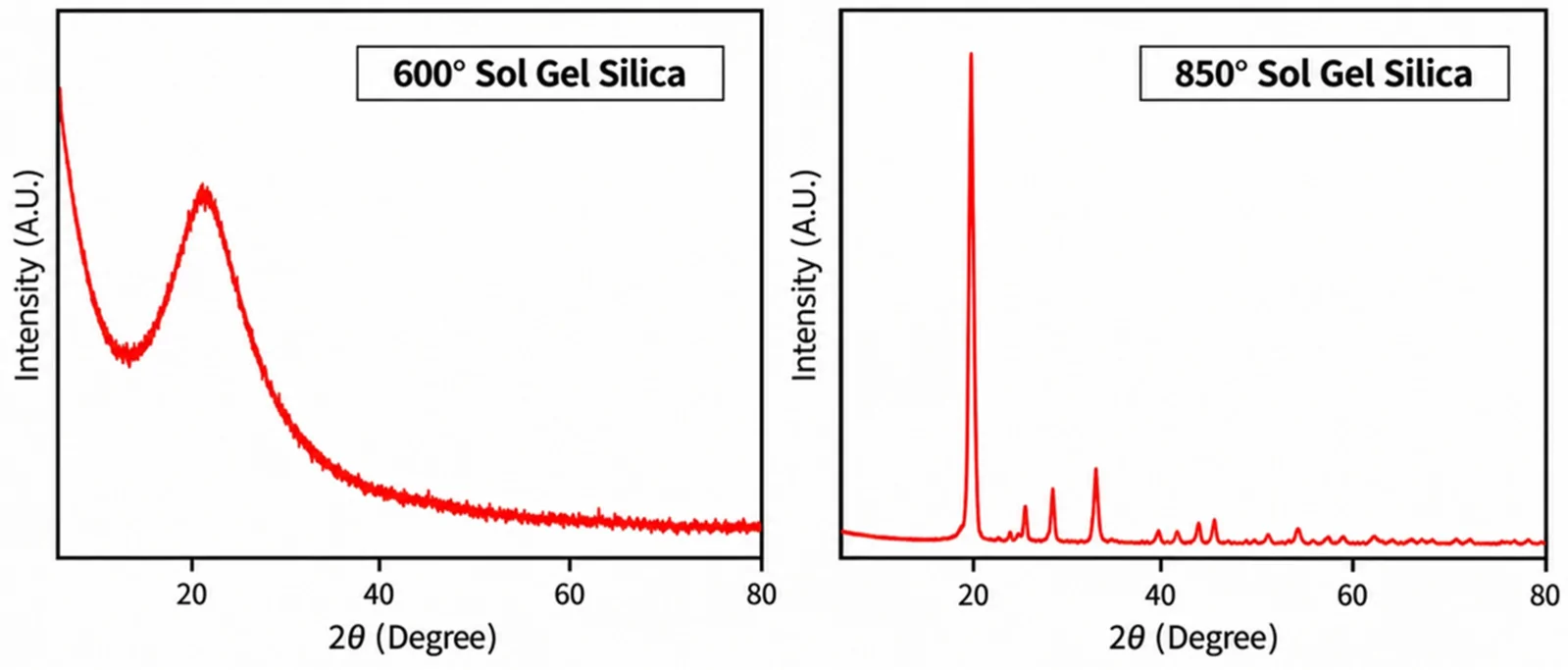
XRD pattern for silica obtained by the sol–gel method with a combustion step performed at 600 °C and 850 °C. Reproduced from Ref. [210] with permission from Springer.

**Figure 16:**
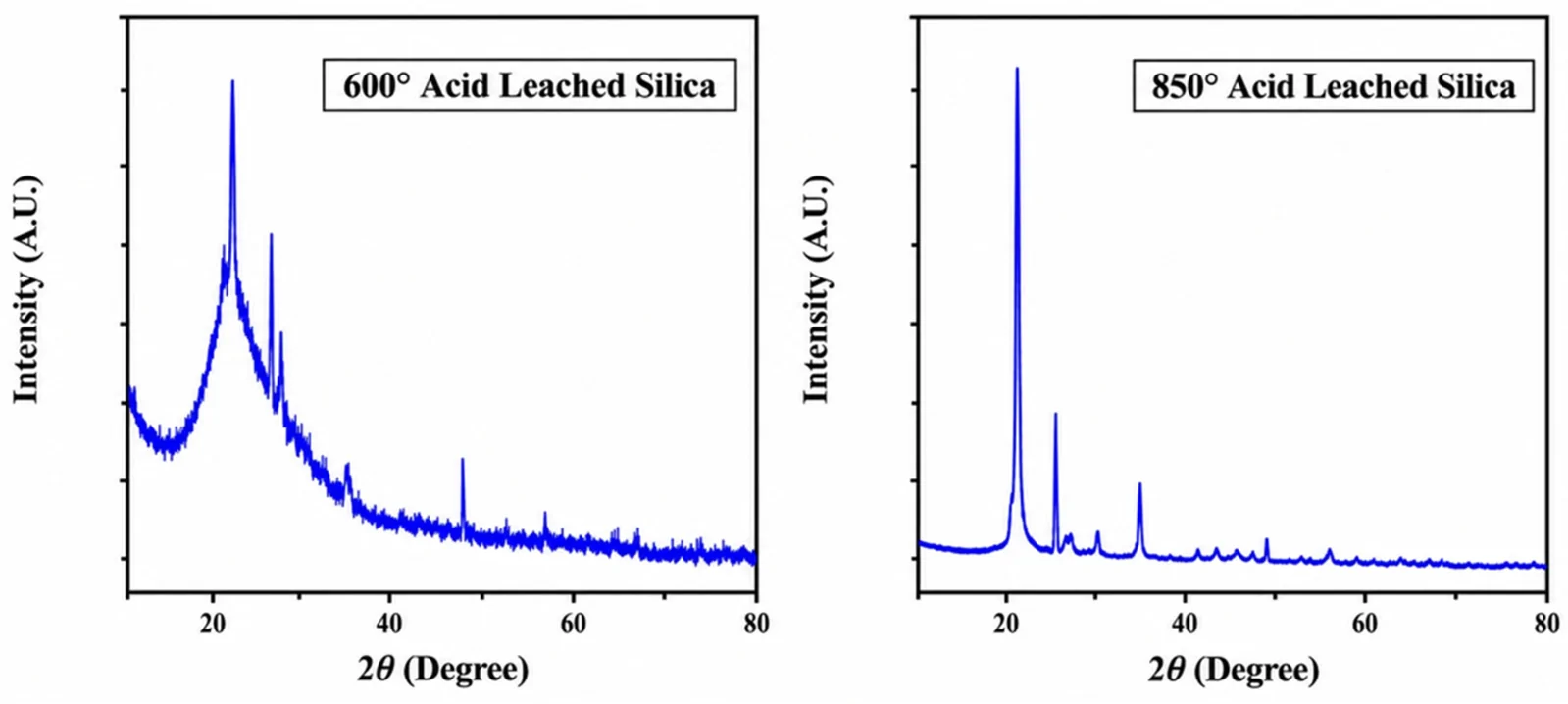
XRD pattern for silica obtained by the acid leaching method with a combustion step performed at 600 °C and 850 °C. Reproduced from Ref. [210] with permission from Springer.

A comparison of materials obtained by different synthesis routes indicates that temperature is a major factor influencing the structure of biosilica. As shown in Figure 15 and Figure 16, silica obtained by the sol–gel and acid-leaching methods exhibits a predominantly amorphous structure at 600 °C, whereas treatment at 850 °C promotes the formation of crystalline phases [82,210]. In contrast, hydrothermal conditions may enable the formation of crystalline structures at considerably lower temperatures because elevated pressure and the aqueous reaction environment facilitate structural reorganization [176,211]. These observations demonstrate that the structural outcome depends not only on temperature itself but also on the applied synthesis route.

### 5.2. Porosity and Specific Surface Area of Silica in Different Synthesis Methods

The data summarized in Table 5 indicate that the investigated synthesis routes can yield materials with substantially different specific surface areas. Sol–gel synthesis can produce both microporous and mesoporous materials, while hydrothermal and acid-leaching approaches may also yield silica with diverse textural characteristics [88,92,136,156,190,206,209,212]. Consequently, no synthesis method can be considered universally superior on the basis of specific surface area or pore size alone. Selection of the synthesis route should instead consider the required combination of structural properties, intended application, process complexity, and environmental burden.

### 5.3. Influence of Synthesis Conditions on Surface Functional Groups

The density and accessibility of surface silanol (Si-OH) groups depend on the conditions used to obtain biosilica. Acid pretreatment facilitates the removal of inorganic impurities, whereas alkaline extraction and subsequent precipitation influence the development of the silica structure and its surface properties [155,165]. Thermal treatment can promote the condensation of neighboring silanol groups into siloxane (Si-O-Si) bridges, leading to a decrease in surface hydroxylation. Consequently, an increase in specific surface area does not necessarily correspond to a higher density of chemically active sites [79].

Optimization of synthesis conditions should therefore consider surface chemistry alongside porosity and purity. FT-IR and solid-state ^29^Si NMR spectroscopy can provide information on silanol groups and the degree of silica network condensation, helping to establish relationships between processing conditions and the availability of reactive surface sites [155].

## 6. Applications of Biosilica

Silica obtained using environmentally friendly methods has a wide range of applications in cosmetics, medicine, agriculture, and environmental remediation [1,24,35,73,101,213,214]. The growing interest in using waste biomass as a source of silica is in line with the circular economy concept, which suggests a potential increase in the importance of this type of material in the future [1,33,214]. However, the potential environmental risks associated with biosilica application, including nanoparticle mobility, contaminant leaching, and long-term ecological effects, must also be considered, as discussed in Section 6.3.

### 6.1. Agriculture

Biosilica is particularly relevant to sustainable agriculture, where advances in environmentally friendly fertilizers and nanotechnology offer new opportunities to improve crop production efficiency. The literature indicates that nanosilica may act as a carrier of nutrients with controlled release, which creates a basis for the development of more advanced fertilization systems adapted to environmental conditions [215,216,217]. Importantly, the agricultural value of waste-derived biosilica is determined not only by silicon supply but also by its material characteristics. Its porous structure and modifiable surface enable biosilica to function as a solid platform whose interaction with nutrients and soil components can be tailored through synthesis and post-treatment. Thus, agricultural-waste-derived biosilica should be considered not merely as a source of plant-available silicon, but as a functional material whose physicochemical properties can be adjusted for specific agricultural applications [67,213,218].

The use of silica nanomaterials obtained by “green synthesis” methods makes it possible to limit nutrient losses, reduce doses of chemical fertilizers and pesticides, and increase the efficiency of their use by plants [218,219,220,221]. Therefore, further development of multifunctional silica materials is anticipated, which may combine fertilizing, protective, and plant-growth-stimulating properties [219,220,221]. Biosilica obtained from agricultural waste may be used to improve soil structure and increase its sorption capacity, which affects the availability and uptake of key nutrients such as carbon, nitrogen, and phosphorus [182,213,218,222].

**Figure 17:**
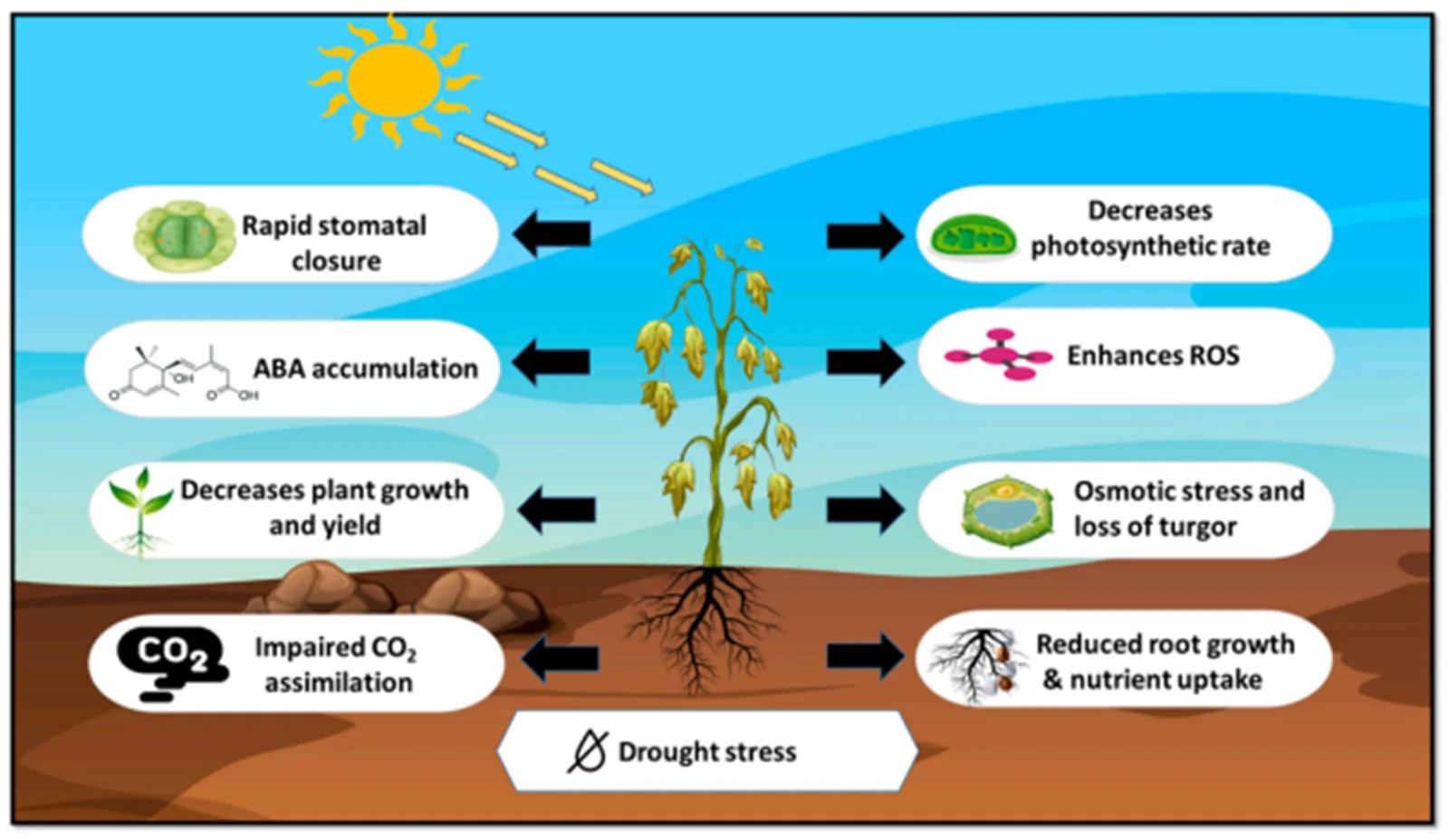
Effect of drought on plants. Reproduced from Ref. [226] under the terms of the Creative Commons Attribution 4.0 License.

Future applications may include the targeted modification of soil physicochemical properties to meet specific cultivation requirements and address challenges associated with climate change [213,218,219,220,221,222]. Thanks to its high specific surface area and developed porosity, silica exhibits the ability to store water, which is of particular importance under drought conditions [223,224]. Its use may lead to increased plant resistance to water stress and limitation of yield losses resulting from water deficiency [224,225,226,227]. In the context of progressing climate change, it is indicated that silica materials may constitute an important element of adaptation strategies in agriculture, especially in regions exposed to water deficit [225,226,227,228,229]. Drought-induced abiotic stress disrupts cell division, limits CO_2_ assimilation, reduces leaf area, impairs photosynthetic activity, induces stomatal closure, and disturbs ionic homeostasis, ultimately decreasing plant productivity (Figure 17) [225,226,227].

In addition, biosilica may support nutrient uptake and contribute to improved plant growth and crop quality [225,230]. Under saline conditions, silicon supplementation may help limit the accumulation of potentially harmful ions, particularly Na^+^ and Cl^−^, in plant tissues [227,230]. One proposed mechanism involves silicon deposition in the root apoplast, which may strengthen apoplastic barriers and reduce the movement of ions into the xylem [227]. Although these effects demonstrate the potential benefits of silicon for plant stress tolerance, their direct applicability to agricultural-waste-derived biosilica requires further investigation.

### 6.2. Hazardous Contaminant Removal and Soil Remediation

In the context of progressive ecosystem degradation driven by intensive industrialization, urbanization, and increasing anthropogenic pressure, the contamination of water and soil resources with heavy metals, metalloids, persistent organic pollutants (POPs), nutrients, pharmaceuticals, dyes, and agrochemicals remains one of the major challenges in contemporary environmental engineering [231]. Conventional treatment technologies based on physicochemical and biological processes are widely applied. However, their efficiency is often constrained by high operational costs, limited selectivity, sensitivity to wastewater matrix composition, and the generation of secondary waste requiring further management [231]. Within this framework, the circular economy paradigm has stimulated increasing interest in adsorbent materials derived from locally available bioresources, including agricultural residues. Biosilica obtained from rice husk, wheat straw, sugarcane bagasse, and other lignocellulosic by-products represents a promising alternative to selected synthetic silica adsorbents produced from mineral raw materials through energy-intensive processes [35,232,233].

In the following discussion, direct performance comparisons are restricted to biosilica, silica derived from agricultural residues, and biosilica-containing composites. Raw agricultural residues, biochars, and activated carbons are cited only as contextual references and are explicitly distinguished from silica-based materials because their surface chemistry and dominant adsorption mechanisms differ.

The potential of biosilica arises from its amorphous structure, developed porosity, reactive surface chemistry, and the possibility of valorizing large-volume agricultural waste streams [79,232,234].

Nevertheless, biosilica should not be considered a uniform class of materials, as its physicochemical properties strongly depend on the biomass source, pretreatment method, combustion or extraction conditions, post-synthetic modification, and particle size distribution [3,79,232]. These factors determine the specific surface area, pore architecture, surface silanol (Si-OH) density, ash purity, and the content of residual inorganic or organic components [79,232]. The contaminant-removal performance of biosilica is therefore closely linked to both its porous architecture and surface chemistry [233,234,235]. In contrast to crystalline silica, amorphous biogenic silica is characterized by a hydroxylated surface containing silanol groups, which may participate in adsorption, ion exchange, surface complexation, hydrogen bonding, and further chemical functionalization [234,235].

These structure–property relationships are directly relevant to remediation performance because the accessibility and chemical nature of active surface sites determine not only adsorption capacity but also contaminant selectivity and immobilization efficiency. Consequently, biosilica performance should be interpreted in relation to both material characteristics and contaminant speciation rather than solely on the basis of maximum adsorption capacity. This is particularly important when comparing materials obtained from different agricultural residues and synthesis routes, for which differences in porosity, surface chemistry, and functionalization may lead to different dominant removal mechanisms.

At the molecular level, metal-ion retention on biosilica surfaces may involve inner-sphere complexation through direct coordination with surface oxygen atoms, as well as outer-sphere associations mediated by electrostatic interactions. The acid–base equilibrium of surface silanol groups and a simplified representation of divalent metal-ion binding can be expressed as follows:

≡Si-OH ⇌ ≡Si-O^−^ + H^+^

≡Si-O^−^ + M^2+^ ⇌ ≡Si-O-M^+^

where M^2+^ represents a divalent metal cation. Sjöberg [236] described the role of silica surface acid–base equilibria and metal-ion complexation in aqueous environments, while Mason et al. [237] provided experimental NMR evidence supporting the formation of inner-sphere complexes involving Cu^2+^ and Ni^2+^ on deprotonated silanol sites of amorphous silica. Nevertheless, the relative contributions of different adsorption pathways depend on metal speciation, solution conditions, and surface characteristics. Therefore, adsorption isotherms and kinetic models alone are insufficient to establish the dominant binding mechanism, and complementary spectroscopic investigations are required for its reliable identification.

Despite the favorable properties of native biosilica, surface modification is frequently required to achieve higher selectivity toward specific contaminants, particularly in complex water and wastewater matrices [235]. One of the most widely used approaches is silanization, which enables the introduction of targeted functional groups onto the SiO_2_ surface. For instance, APTES (3-aminopropyltriethoxysilane) can form Si-O-Si linkages with surface silanols and introduce amino groups. Under acidic or near-neutral conditions, protonation of these amino groups may generate positively charged sites, thereby enhancing the adsorption of anionic dyes and oxyanions [238]. Such modification may improve the removal of anionic dyes, including methyl orange and Congo red, and may also support the binding of selected metal ions through complexation, chelation, and pH-dependent electrostatic interactions [235].

Another important modification pathway involves the incorporation of natural polymers, such as chitosan and alginates, or synthetic polymers, including polyethyleneimine (PEI) and polyaniline (PANI). These hybrid systems combine the structural stability of the SiO2 matrix with a high density of polymer-derived binding sites [235]. Promising results have also been reported for biosilica hybrids with reduced graphene oxide (rGO).

Their synthesis may promote the formation of Si-O-C bridges, interfacial polarization, and heterogeneous active sites associated with structural defects [238]. In such systems, adsorption may involve π-π interactions, cation-π interactions, hydrogen bonding, and donor-acceptor interactions, which are particularly relevant for aromatic organic contaminants and dyes [238].

The incorporation of magnetic nanoparticles, such as Fe_3_O_4_, into silica-based matrices enables the separation of the adsorbent from treated water using an external magnetic field. This strategy may facilitate material recovery and regeneration. However, its practical applicability depends on synthesis cost, magnetic stability, resistance to iron leaching, regeneration efficiency, and the number of feasible reuse cycles [233,239]. Functionalization of biosilica with amino acids, such as L-cysteine, provides another route to introduce thiol, amino, and carboxyl groups. Such surfaces may exhibit zwitterionic character and high affinity toward Hg(II) species, mainly due to sulfur and nitrogen donor atoms. Materials of this type may retain sorption capacity even under acidic conditions, for example at pH ≈ 3, although their stability and selectivity should be verified individually in relevant environmental matrices [240].

The main surface active sites and their interactions with different classes of environmental contaminants are summarized in Table 6.

#### 6.2.1. Removal of Inorganic Contaminants from Water and Wastewater

##### Heavy Metals

Due to the tunability of its surface properties, biosilica can be applied in the treatment of surface water, groundwater, and industrial wastewater. The adsorption of metal ions on silica-based materials occurs mainly through surface complexation, ion exchange involving protons from silanol groups, and electrostatic interactions controlled by pH, ionic strength, competing ions, and dissolved organic matter [234]. Therefore, adsorption capacities obtained in single-solute model systems should be interpreted cautiously, as real wastewater matrices may substantially alter sorption performance through competition, fouling, or changes in metal speciation. For comparison, non-silica agricultural adsorbents, including corn-straw and municipal-sludge-derived biochars [244] and raw rice husk biosorbent [245], have also been investigated for heavy-metal removal. However, their adsorption mechanisms are governed largely by carbonaceous and biomass-derived surface functionalities and are therefore not directly comparable with biosilica.

Lead ions (Pb^2+^) can exhibit considerable affinity for silica-based adsorbents, depending on their surface chemistry and solution conditions. In studies on amphoteric polymer scaffolds containing biosilica (ACS-4B), an adsorption capacity of 539 mg/g was achieved [246]. Rice-husk-derived silica showed adsorption capacities of 270.63, 52.14, and 21.69 mg/g for Pb^2+^, Ni^2+^, and Fe^3+^, respectively, in battery wastewater [247], while a later study using rice-husk-derived silica nanoparticles reported capacities of 44.93 mg/g for Pb^2+^ and 37.91 mg/g for Cd^2+^, with only 6–7% capacity loss after seven regeneration cycles [248].

Chromium occurs in industrial wastewater mainly in two oxidation states: Cr(III), which is generally less toxic and less mobile, and Cr(VI), which is highly toxic and carcinogenic and may exhibit greater mobility depending on environmental conditions. Non-silica agricultural adsorbents have also been investigated for chromium removal. Wheat and corn straw showed adsorption capacities of 125.6 and 87.4 mg/g for Cr^6+^, respectively [249], while activated carbon produced from sugarcane bagasse has also been applied to Cr^6+^ removal [250].

These examples are retained only as contextual comparisons and are not included in the direct assessment of biosilica performance because their adsorption mechanisms arise predominantly from lignocellulosic or carbonaceous surface functionalities. In addition, adsorption processes requiring strongly acidic conditions may have limited practical applicability because of increased operating costs, corrosion risks, the need for post-treatment neutralization, and secondary waste generation. The maximum adsorption capacities reported for selected biosilica and biosilica-containing materials are summarized in Table 7. Direct comparison is restricted to materials in which silica constitutes the adsorbent matrix or a defined functional component, thereby avoiding comparison with raw biomass and conventional biochars having fundamentally different surface chemistries.

##### Metalloids and Oxyanions

For the removal of nutrient pollutants, silica-based materials modified with metal ions and zeolitic materials synthesized from rice husk ash are of particular interest. For example, Fe-ZSM-5 obtained from silica extracted from rice husk ash demonstrated the ability to adsorb ammonium and phosphate ions, indicating the potential of such materials for mitigating eutrophication [234]. In the case of arsenic, it is necessary to distinguish between As(III) and As(V), as these species differ markedly in speciation, charge, mobility, and affinity toward adsorbent surfaces. As(V) usually occurs as anionic species, whereas As(III), at near-neutral pH, may occur predominantly as a weakly dissociated or neutral species. Consequently, surface modification with iron, aluminum, or manganese oxides is commonly applied to enhance arsenic removal, and in some systems a prior oxidation step converting As(III) to As(V) may be required [234,235].

#### 6.2.2. Removal of Organic Contaminants from Aquatic Systems

##### Textile Dyes

Dye pollutants originating from the textile industry constitute an important environmental problem because many synthetic dyes exhibit toxicity, persistence, complex molecular structures, and limited biodegradability.

Functionalized biosilica enables dye removal through multiple mechanisms, including hydrophobic interactions, hydrogen bonding, electrostatic interactions, and, in systems containing aromatic structures, π-π interactions [235]. The stability of adsorbents during regeneration cycles depends on the nature of the contaminant, the type of functionalization, and the eluent used. Literature data indicate that selected silica adsorbents and nanoadsorbents may retain high performance after several regeneration cycles and may be suitable for repeated use when appropriate washing strategies are applied, including pH adjustment, salt solutions, solvents, or complexing agents [235,252]. Nevertheless, regeneration efficiency should be evaluated together with possible loss of functional groups, desorption of retained contaminants, eluent toxicity, and the management of spent regenerant solutions. Moreover, repeated-use studies should include possible silica dissolution, leaching of incorporated metals or surface modifiers, changes in adsorption selectivity, and contaminant release during successive regeneration cycles, particularly under environmentally relevant pH, ionic-strength, and dissolved-organic-matter conditions.

Dyes such as methylene blue (MB, cationic) and Congo red (CR, anionic) can be removed simultaneously using advanced silica-based adsorbents. Amphoteric composite materials, such as ACS-4B, exploit the presence of -SiOH, -COOH, and -NH_2_ groups to regulate surface charge as a function of solution pH [246]. For contextual comparison, untreated or processed lignocellulosic materials such as sugarcane bagasse pulp have also shown high methylene-blue removal efficiency [253]. However, these materials are not included in the direct comparison of biosilica performance because adsorption on lignocellulosic surfaces is governed by a different set of functional groups and structural features.

Dye adsorption kinetics are frequently described using the pseudo-second-order model; however, a good fit to this model does not, by itself, establish the underlying adsorption mechanism or identify the rate-controlling step [253].

##### Pharmaceuticals and Antibiotics

The increasing occurrence of pharmaceutical residues in surface waters necessitates the development of efficient methods for their extraction, immobilization, or degradation. Biosilica and biosilica-derived composites show potential in this field due to their tunable surface chemistry, but their selectivity and performance are strongly compound-specific and matrix-dependent.

Silica nanoparticles (SNPs) synthesized from sugarcane bagasse have been successfully applied for tetracycline removal, with optimal performance achieved at pH 7.0 [254]. Other agricultural-waste-derived materials, including N,S-codoped magnetic bagasse biochar [255] and biochars obtained from cotton-gin waste and guayule bagasse [256], have also been investigated for pharmaceutical adsorption. 

These materials are mentioned only as contextual examples because their adsorption behavior is dominated by carbonaceous surface chemistry and cannot be directly extrapolated to biosilica. Biochars derived from cotton and guayule waste have also demonstrated the ability to remove sulfapyridine (SPY), docusate (DCT), and erythromycin (ETM) [256]. Broader reviews of agricultural-waste-derived adsorbents have proposed parameters such as the Drug Mass Removal Rate (DMRR) for comparing pharmaceutical removal by non-silica biosorbents [257]. Activated carbons and other carbonaceous materials derived from agricultural residues have also been investigated for pharmaceutical and related organic contaminant removal [257,258]. However, because these materials differ fundamentally from biosilica in composition and adsorption mechanism, their quantitative performance is not directly compared with biosilica in the present review.

Therefore, the limited number of studies directly investigating agricultural-waste-derived biosilica for the removal of pharmaceuticals and antibiotics represents an important research gap.

##### Pesticides and Agrochemicals

Direct evidence for pesticide removal using agricultural-waste-derived biosilica remains limited. Raw agricultural residues, including eucalyptus bark, corn cobs, and rice husks, have been investigated as sorbents for atrazine and imidacloprid [259], while biochar has also been evaluated as a soil amendment in systems containing glyphosate [260]. However, these materials are not biosilica, and their adsorption behavior is strongly influenced by lignocellulosic or carbonaceous domains, aromaticity, surface polarity, and organic functional groups. Therefore, they are retained here only as contextual evidence and are not directly compared with biosilica. This distinction highlights the need for dedicated studies evaluating the contribution of silanol groups, silica porosity, and surface functionalization to pesticide adsorption by agricultural-waste-derived biosilica.

##### Persistent Organic Pollutants

Persistent organic pollutants (POPs), including organochlorine pesticides, polychlorinated biphenyls (PCBs), and dioxins, represent a global environmental threat due to their persistence, toxicity, bioaccumulation potential, and biomagnification in trophic chains [239,242,261]. Conventional wastewater treatment methods, such as coagulation and activated sludge processes, may be insufficient for some POPs, especially at low concentrations, under conditions of high hydrophobicity, or in the presence of complex organic matrices [239]. Silica nanomaterials, particularly after modification aimed at increasing hydrophobicity or donor-acceptor affinity, may support POPs removal through van der Waals forces, hydrophobic interactions, π-π interactions, and donor-acceptor interactions [242,243]. The integration of biosilica with advanced oxidation processes (AOPs) may expand its function from adsorption alone to supporting the degradation or mineralization of persistent organic compounds. However, in such systems, the formation and toxicity of transformation products must be carefully monitored, as partial degradation does not necessarily correspond to complete detoxification [262].

#### 6.2.3. Soil Remediation Using Biosilica and Nanosilica

Soil is a complex matrix in which contaminants occur in various chemical forms that determine their mobility, bioavailability, and toxicity to plants and microorganisms. The application of biosilica and nanosilica in soil remediation is primarily aimed at reducing heavy metal mobility through adsorption, immobilization, and changes in chemical speciation [241,263]. The addition of nanosilica to contaminated soil may initiate several physicochemical processes. First, nanosilica may affect soil pH. In selected experimental systems, nanosilica application has been associated with an increase in soil pH, which may reduce the solubility of some metals through sorption, precipitation, or formation of less mobile species. However, this effect depends on soil type, buffering capacity, carbonate content, organic matter, and material dosage [262,264].

Second, metals such as Pb, Cu, and Zn may bind to silica surfaces or form surface complexes and poorly soluble silicate species, thereby reducing their bioavailability [241,264]. Third, nanosilica may alter metal speciation. Sequential extraction studies have shown that nanosilica applied at doses of approximately 1000 mg/kg may decrease the proportion of metals in exchangeable fractions while increasing their association with Fe-Mn oxides, organic matter, and residual fractions [231]. At a nanosilica dose of 1000 mg/kg, the exchangeable fractions of Pb, Cu, Zn, Ni, and Cr decreased by 4.5–8.0% relative to the control, indicating the potential of nanosilica to reduce metal mobility in contaminated soils [231].

Importantly, this type of remediation primarily decreases contaminant mobility and bioavailability rather than the total contaminant concentration in soil [231,241]. The immobilization effect should not be regarded as necessarily permanent, because changes in pH, redox conditions, ionic composition, or organic matter may modify contaminant speciation and surface interactions, potentially leading to partial remobilization [231,241,263]. Therefore, long-term stability should be evaluated under variable environmental conditions rather than inferred solely from short-term decreases in mobile or extractable contaminant fractions. Moreover, silica nanoparticles may influence soil microbial communities, either directly through nanoparticle–microorganism interactions or indirectly through changes in soil chemistry, nutrient availability, and contaminant bioavailability. For example, Tian et al. demonstrated that SiO_2_ nanoparticle application altered soil metabolite profiles and bacterial and fungal community composition in the rhizosphere of pakchoi grown in contaminated mine soil [264]. Thus, assessment of microbial community structure and activity should be included in future long-term studies of biosilica-assisted soil remediation.

### 6.3. Limitations and Potential Environmental Risks of Biosilica Applications

Despite its potential benefits, biosilica application may also involve environmental risks that depend on soil properties, material characteristics, and application rates. Changes in soil water retention and nutrient dynamics may influence water availability and nutrient transport under specific environmental conditions. Therefore, the long-term effects of biosilica application on soil hydrology and nutrient mobility should be evaluated under field conditions [223].

Additional risks may arise from the composition and physicochemical form of the biosilica itself. Residual inorganic or organic contaminants originating from the agricultural feedstock or introduced during processing may remain in the final material, while surface modifiers or incorporated metal-containing phases may potentially undergo leaching during environmental exposure. In the case of nanoscale biosilica, particle mobility, transformation, and interactions with biological components of soil should also be considered. Importantly, high adsorption or immobilization efficiency should not automatically be interpreted as reduced ecological risk, particularly when material ageing or changing environmental conditions affect contaminant retention [231,241,260,264].

Therefore, environmental safety assessments should complement conventional adsorption-performance testing and include the evaluation of material purity, leaching behavior, ecotoxicity, long-term stability, and the environmental fate of nanoscale fractions.

## 7. Challenges, Research Gaps and Future Directions

### 7.1. Limitations of Current Biosilica Research

Despite the dynamic development of research on biosilica synthesis from agricultural waste, the current state of knowledge indicates that these technologies are still at a transitional stage between basic research and potential industrial implementation [32,75,265].

One of the main limitations is the fragmented nature of available studies, which usually focus on selected aspects of the process, such as biomass type or individual synthesis parameters, without integrating them into a comprehensive framework. As a result, it is difficult to unequivocally determine optimal biosilica production strategies depending on its target application.

A significant challenge remains the high variability of biomass chemical composition, resulting from environmental, species-related, and agronomic differences. This variability directly affects the quality and reproducibility of the resulting materials, making it difficult to control their physicochemical properties. Consequently, synthesis processes often require additional purification stages, which increases their complexity and limits their scaling potential [38,79].

Despite extensive research on biomass-derived silica, several important knowledge gaps remain. In particular, quantitative evidence linking specific surface characteristics to contaminant selectivity and removal mechanisms is still insufficient. The effects of competing ions and organic matter on adsorption behavior have not been systematically evaluated across different biosilica materials. Furthermore, comparative studies addressing regeneration efficiency, contaminant desorption, and changes in surface properties during repeated adsorption cycles remain limited. These gaps restrict the assessment of biosilica performance under environmentally relevant conditions and hinder the identification of materials suitable for specific remediation applications.

### 7.2. Challenges for Scale-Up and Environmental Implementation

Another key challenge is process scalability. Most studies are carried out under laboratory conditions that do not reflect the complexity of industrial processes.

Scaling up these technologies requires optimization of energy consumption, reagent use, and processing time while maintaining consistent product quality. In this context, it becomes particularly important to take economic aspects and material efficiency into account [265].

Paradoxically, although biosilica synthesis is often described as “green”, these processes may still require the use of strong chemical reagents, which generate additional environmental burdens. This points to the need for a more critical assessment of the actual sustainability of these technologies, taking into account the full life cycle of the material [32,75].

In the context of agricultural applications, the shortage of long-term studies and experiments conducted under field conditions is particularly visible. Current results, based mainly on laboratory and greenhouse studies, do not fully reflect the complexity of soil–plant systems. This limits the possibility of assessing the actual effectiveness of biosilica and its impact on plant productivity and soil properties over the longer term [226,230].

An equally important research gap is the limited understanding of the long-term environmental impacts of biosilica. It remains particularly important to understand its mobility, transformations, and interactions with soil microorganisms. Increased water retention and changes in nutrient availability may lead to unintended environmental effects that require further investigation [223,224]. From the application perspective, there is also a clear shortage of comprehensive life-cycle and techno-economic assessments covering feedstock collection, reagent and water consumption, energy demand, waste generation, regeneration, and end-of-life management. Without such quantitative analyses, claims regarding the sustainability, economic competitiveness, or suitability of biosilica technologies for large-scale implementation should remain cautious.

### 7.3. Future Research Perspectives

Although pilot-scale production and field applications of silica-based materials have already been investigated, further research on agricultural-waste-derived biosilica should focus on the molecular mechanisms governing contaminant adsorption and immobilization. Particular attention should be given to the influence of surface silanol groups, pore accessibility, and contaminant speciation on adsorption selectivity. Combining advanced spectroscopic techniques, such as FT-IR, XPS, and X-ray absorption spectroscopy, with molecular modeling could provide deeper insight into surface complexation and competitive adsorption in multicomponent systems [231,235,241,263].

Another important direction is elucidating the mechanisms responsible for contaminant remobilization during changes in pH, ionic strength, and redox conditions, including the role of silica dissolution and surface transformations. Investigating these processes alongside biosilica-microorganism interactions could support the development of predictive structure–property–performance relationships and the design of materials tailored to specific environmental applications [231,241,264].

## 8. Conclusions

Agricultural residues offer promising opportunities for biosilica production, combining waste valorization with environmental remediation. The reviewed studies demonstrate that feedstock composition and synthesis conditions determine the physicochemical properties of the resulting materials, while contaminant-specific surface interactions govern their removal efficiency. Although biosilica shows potential for water treatment and soil remediation, its practical implementation remains limited by feedstock variability, insufficient validation under realistic environmental conditions, and uncertainties regarding regeneration and long-term stability. Future research should therefore focus on reproducible synthesis, performance evaluation in complex environmental matrices, and comprehensive environmental and techno-economic assessments. Addressing these challenges will be essential for developing biosilica-based remediation technologies that are effective, reliable, and sustainable.

## Figures and Tables

**Table 1:** Reported SiO_2_ contents in selected agricultural and plant-derived feedstocks on different analytical bases.

Plant Material	Reported Parameter	Reported Content [%]	Basis/Material Analyzed	Ref.
Rice husk	SiO_2_	93.2	Ash	[81]
Rice straw	SiO_2_	75.0	Ash	[82]
Corn cob	SiO_2_	50.0–60.0	Ash	[70,80]
Corn ears	SiO_2_	64.0	Ash	[70]
Sugarcane bagasse	SiO_2_	81.6	Ash	[1]
Sugarcane leaves	SiO_2_	80.1	Ash	[83]
Wheat husk and spike	SiO_2_	86.0	Ash	[132]
Banana peel	SiO_2_	0.35	Biomass/plant material	[72,117]
Potatoes	Si	up to 0.08 (leaves); up to 0.30 (roots)	Dry biomass	[68]

**Table 2:** Drying conditions of materials after washing with distilled water.

Drying Method	Drying Conditions	Ref.
Freeze-drying	3 h, −50 °C	[139]
Vacuum drying	1 h, from 0 °C to 100 °C, 0.5 atm	[141]
Convective drying	4 days, RT + 2 h, 100 °C	[142]
Convective drying	1 h, 120 °C	[143]
Convective drying	24 h, 105 °C	[144,145]
Convective drying	24 h, 110 °C	[146]
Convective drying	2 h, 120 °C	[147]

**Table 3:** Summary of reagents and reaction conditions used for biosilica synthesis in the sol–gel method.

Material	Ash Production	Reaction Conditions with Base	Acid	Final pH Value	Drying	Environmental Considerations	Ref.
Cassava tuber peels	3 h, 500 °C	2 M NaOH, 3 h, 80 °C	1 M HCl	7.0	100 °C	Calcination; NaOH and HCl consumption; washing effluent	[143]
Rice husks	4 h, 650 °C	1 M NaOH, 2 h, 80 °C	1 M HCl	7.0	24 h, 110 °C	Prolonged calcination and drying; acid/alkali use; washing effluent	[182]
Corn cobs	500 °C	NaOH, 1 h, 80 °C	1 M HCl	7.0	20 h, 700 °C	High-temperature drying; NaOH/HCl use; energy demand	[107]
Rice husks	2 h, 700 °C	2.5 M NaOH, 2 h, 100 °C	1 M HCl	7.0	8 h, 200 °C	High-temperature calcination; concentrated alkali; prolonged drying	[183]
Rice husks	-	NaOH, 15–30 min, 110–120 °C, 0.15–0.2 MPa	1 M HCl	7.0	20 h, 60 °C	Elevated temperature/pressure; acid/alkali consumption; long drying	[184]
Palm kernel shells	3 h, 750 °C	3 M NaOH, 2 h	3 M HCl	7.5–8.5	24 h, 80 °C	High-temperature calcination; concentrated acid/alkali; long drying	[185]
Sugarcane	4 h, 650 °C	1 M NaOH, 1 h, 90 °C	1 M HCl	7.0	24 h, 100 °C	Calcination and prolonged drying; acid/alkali use; organic-acid alternatives	[186]
			5% citric acid				
			5% L-cysteine hydrochloride monohydrate				
Sugarcane bagasse	7 h, 900 °C	1 M NaOH	6 M H_2_SO_4_	7.0	24 h, 80 °C	Very high calcination temperature; concentrated H_2_SO_4_; high energy and chemical demand	[187]
Wheat husks	700 °C	NaOH, 1 h, 80 °C	1 M HCl	7.0	20 h, 700 °C	High-temperature thermal treatment; acid/alkali use; energy demand	[188]

Environmental considerations are qualitative and are based on the reported process conditions, including thermal treatment, reagent use, pressure, and process duration.

**Table 4:** Summary of reagents and reaction conditions used in the acid leaching method.

Material	Acid	Reaction Conditions	Washing (pH)	Drying	Combustion	Environmental Considerations	Ref.
Rice husks	10 wt.% H_2_SO_4_	3 h/80 °C	7.0	3/50 °C	4 h/800 °C	Mineral-acid consumption; washing effluent; high-temperature calcination	[114,115]
Sugarcane bagasse	5 wt.% citric acid	24 h/80 °C	6.5	overnight at 40 °C	4 h/700 °C	Organic acid; very long leaching; calcination energy demand	[64]
Rice husks	0.1 M HCl	24 h/RT	7.0	–	2 h/800 °C	Long leaching; HCl consumption; high-temperature calcination	[116]
Rice husks	3 M HCl	4 h/80 °C	–	6 h/100 °C	4 h/800 °C	Concentrated HCl; prolonged heating; high calcination demand	[117]
Oil palm	6% citric acid	1 h/70 °C	–	1 h/60 °C	30 min/800 °C	Organic-acid route; relatively short processing; high calcination temperature	[109]
Rice husks	3 M HCl/3 M H_2_SO_4_/3 M HNO_3_	RT	7.0	24 h/50 °C	2 h/600 °C	Concentrated mineral acids; washing effluent; long drying	[118]
Rice husks	10% HCl	2 h	–	24 h/150 °C	2 h/700 °C	HCl consumption; prolonged high-temperature drying; calcination energy demand	[119]

Environmental considerations are qualitative and refer to the principal process burdens associated with the reported synthesis conditions.

**Table 5:** Comparison of representative biosilica production routes in terms of material properties, process requirements, and environmental limitations.

Route/Feedstock	Reported Properties	Main Reagents and Processing Conditions	Key Limitations	Ref.
Sol-gel Teff straw	Yield: n.r. Purity: n.r. BET: 807.163 m^2^/g	Acid and alkali; water washing required (volume n.r.) Thermal treatment required (temperature n.r.)	Acid/alkali use; washing effluent; drying and thermal energy	[206]
Sol-gel Rice husk	Yield: n.r. Purity: n.r. BET: n.r.	Acid and NaOH; washing required (volume n.r.) Thermal treatment and drying required	Chemical consumption; washing effluent; drying	[34]
Microwave-assisted sol-gel Rice husk	Yield: n.r. Purity: n.r. BET: 400–700 m^2^/g	Alkali and acid; water use n.r. Microwave-assisted processing at elevated temperature	Reagent use; wastewater generation; thermal/microwave energy demand	[209]
Sol-gel Rice straw ash	Yield: ~76% Purity: n.r. BET: n.r.	2.5 M NaOH and 2 M H_2_SO_4_; distilled-water washing (volume n.r.) Extraction: 100 °C; drying: 80 °C	Acid/alkali use; prolonged reaction; washing effluent	[89]
Hydrothermal Rice husk ash	Yield: n.r. Purity: n.r. BET: n.r.	NaOH and 1 N HCl; distilled-water washing (volume n.r.) 110–120 °C; 0.15–0.2 MPa; 15–30 min	Pressurized heating; alkali use; washing and drying	[184]
Hydrothermal Rice husk ash	Yield: n.r. Purity: n.r. BET: n.r.	Fe_2_O_3_-assisted system; water use n.r. Hydrothermal conditions n.r.	Pressure and thermal-energy requirements; reagent use	[195]
Acid leaching Rice hull	Yield: n.r. Purity: n.r. BET: n.r.	Acid; washing required (volume n.r.) Subsequent thermal treatment; typically ~600–700 °C	Acid use; washing effluent; thermal treatment	[88]
Acid leaching Rice husk	Yield: n.r. Purity: n.r. BET: 234.25 m^2^/g	Acid; washing required (volume n.r.) Thermal treatment required (temperature n.r.)	Acid use; washing effluent; calcination	[136]
Acid leaching Rice straw/horsetail	Yield: n.r. Purity: 99.7% BET: 303 m^2^/g	Citric acid; 1300 mL water per 100 g feedstock Thermal conditions n.r. in the cited example	Acid and water consumption; washing; drying/calcination energy	[156]
Acid leaching Sugarcane biomass	Yield: n.r. Purity: 99.7% BET: 323 m^2^/g	7 wt.% citric acid + H_2_SO_4_; 1200 mL water per 50 g feedstock, plus washing Multistage calcination: 310 → 450 → 560 → 600 °C	High water use; acid consumption; multistage heating and drying	[138]

Note: n.r.—not reported.

**Table 6:** Surface active sites and their interactions with environmental contaminants.

Active Sites	Contaminants	Interaction Mechanism	Ref.
Si–OH, Si–O^−^	Pb^2+^, Cu^2+^, Ni^2+^	Surface complexation; electrostatic interactions	[231,236,237,241]
–NH_3_^+^	Anionic dyes	Electrostatic attraction	[235]
–SH, –NH_2_, –COOH	Hg(II) and selected metal ions	Coordination; chelation	[240]
Metal-oxide surface sites (Fe, Al, Mn)	Arsenic oxyanions	Surface complexation; ligand exchange	[235]
Aromatic sites in silica–rGO hybrids	Aromatic dyes	π-π interactions; hydrogen bonding	[238]
Hydrophobic surface groups	Persistent organic pollutants	Hydrophobic interactions; van der Waals forces	[242,243]

**Table 7:** Comparison of maximum adsorption capacities (Q_max_) of selected biosilica and biosilica-containing materials for heavy-metal removal.

Adsorbent	Metal	Q_max_ (mg/g)	Optimum pH	Isotherm Model	Ref.
ACS-4B scaffold	Pb^2+^	539.0	2.9	n.d.	[246]
ACS-4B scaffold	Cr	585.0	2.9	n.d.	[246]
SiO_2_NPs@25	Pb^2+^	270.63	8.0	Freundlich	[247]
SiO_2_NPs@25	Fe^3+^	21.69	8.0	Freundlich	[247]
SiO_2_NPs@25	Ni^2+^	52.14	8.0	Freundlich	[247]
Rice husk silica-modified sludge-based biochar	Pb^2+^	230.5	n.d.	n.d.	[251]

Note: n.d.—not determined.

## Data Availability

No new data were created or analyzed in this study. Data sharing is not applicable to this article.

## Abbreviations

The following abbreviations are used in this manuscript:

AOPs	Advanced oxidation processes
APTES	3-Aminopropyltriethoxysilane
BET	Brunauer–Emmett–Teller
CR	Congo red
DCT	Docusate
DMRR	Drug mass removal rate
ETM	Erythromycin
FE-SEM	Field-emission scanning electron microscopy
IUPAC	International Union of Pure and Applied Chemistry
LSI	Low silicon transporter
MB	Methylene blue
n.d.	Not determined
n.r.	Not reported
PANI	Polyaniline
PCBs	Polychlorinated biphenyls
PEI	Polyethyleneimine
POPs	Persistent organic pollutants
rGO	Reduced graphene oxide
RHA	Rice husk ash
RT	Room temperature
SEM	Scanning electron microscopy
SNPs	Silica nanoparticles
SPY	Sulfapyridine
TEM	Transmission electron microscopy
XRD	X-ray diffraction
ZSM-5	Zeolite Socony Mobil-5
